# Exploratory analysis of choriocapillaris vasculature as a biomarker of idiopathic epiretinal membrane

**DOI:** 10.1371/journal.pone.0306735

**Published:** 2024-07-05

**Authors:** Gee-Hyun Kim, Jiho Lee, Young-Hoon Park

**Affiliations:** 1 Department of Ophthalmology, Seoul St. Mary’s Hospital, College of Medicine, The Catholic University of Korea, Seoul, Republic of Korea; 2 Catholic Institute for Visual Science, College of Medicine, The Catholic University of Korea, Seoul, Republic of Korea; Medizinische Universitat Graz, AUSTRIA

## Abstract

**Purpose:**

To investigate the preoperative choriocapillaris perfusion (CCP) as a biomarker in patients with idiopathic epiretinal membrane (iERM).

**Materials and methods:**

28 patients (28 eyes) with unilateral iERM who received pars plana vitrectomy (PPV) with internal limiting membrane (ILM) peeling were included for retrospective observational study. Optical coherence tomography (OCT) and angiography (OCTA) was performed before and after PPV. Area, perimeter, and circularity of superficial foveal avascular zone (FAZ) were analyzed preoperatively in both eyes using OCTA. Preoperative CCP was also analyzed with binarized en-face OCTA images. Measurements of best-corrected visual acuity (BCVA) and central foveal thickness (CFT) by OCT were conducted at the baseline and 6 months following the surgery. The correlations of preoperative OCT parameters with postoperative BCVA and CFT reduction were analyzed.

**Results:**

CCP was significantly lower (*p* < 0.001) and FAZ had shrunk (*p* < 0.001) in eyes with iERM compared to unaffected fellow eyes before surgery. BCVA and CFT became significantly improved after surgery (*p* = 0.001, *p* < 0.001). Multiple regression analysis revealed that preoperative CCP was significantly related with BCVA improvement (β = 0.185, *p* = 0.005), postoperative BCVA (β = 0.108, *p* = 0.023) and ratio of post- to preoperative CFT (β = 0.106, *p* = 0.044).

**Conclusions:**

Preoperative CCP is a biomarker for poor functional and anatomical prognosis after surgery in iERM.

## Introduction

Idiopathic epiretinal membrane (iERM) is a widespread retinal disease. Proliferation of fibers and cells on the internal limiting membrane (ILM) results in the formation of an ERM, which causes distortion of the macula’s structure, potentially leading to metamorphopsia and/or a decline in visual acuity [1]. The prevalence of ERM in Asian populations has been documented at 12.1%, with the rate varying based on age and axial length [1].

Pars plana vitrectomy, combined with ERM removal, is the primary treatment approach aimed at reducing traction on the retina to improve both morphology and functionality. To prevent the recurrence of ERM, it is common practice to perform concurrent peeling of the ILM [2]. Even with morphological recovery following surgical intervention, patients may experience persistent visual disturbances. Hence, numerous preoperative factors, including preoperative inner retinal deformation, have been investigated to predict the visual outcome following surgery [3].

Optical coherence tomography (OCT) has emerged as a crucial diagnostic tool for macular diseases, starting from the early 1990s. By enabling precise and comprehensive discovery of retinal structures, it unveils the correlation between visual function and the outer retinal layer deformity. As prognostic factors for ERM, the initial proposal focused on the structural alterations of the ellipsoid zone and interdigitation in the foveola [4–8]. Furthermore, OCT has been employed to investigate the relationship between metamorphopsia and structural changes caused by ERM traction, such as the augmentation of an ectopic inner retinal layer in the fovea [9–13], and inner retinal changes impacting the pathway of blood vessels [10–14]. While OCT has uncovered the connection between visual disturbances associated with ERM and retinal structural changes, there is currently a lack of consensus regarding the staging of ERM and the optimal timing for ERM surgery [12].

Enhanced depth imaging (EDI) methods in OCT even enabled observation of the choroid in vivo. Although several studies have investigated choroidal vascular index (CVI) and choroidal thickness in iERM, the results are conflicting [15–18]. Presently, OCT angiography (OCTA) has gained recognition as a non-invasive technique for assessing retinal and choroidal vessels. Due to its tomographic nature, it visualizes vascular plexuses layer by layer, such as choriocapillaris vasculature. Recent studies have reported reduction of choriocapillaris vasculature in iERM eyes compared to unaffected fellow eyes, and this reduction is reversible following surgery [19–21].

The utilization of OCTA is also valuable for the measurement of the foveal avascular zone (FAZ) [22, 23], which represents retinal deformity in ERM eyes. It has revealed an increase in foveal vascular density and a decrease in parafoveal vascular density in eyes with ERM prior to surgery, and conversely, these patterns were observed to reverse after surgery [24, 25]. Several recent studies utilizing OCTA have documented that FAZ enlarges and transforms into a more circular shape following surgery in eyes affected by ERM [25–28].

Using OCTA in this study, we analyzed choriocapillaris perfusion (CCP) and superficial FAZ before ERM surgery. This study aims to investigate their association with functional and anatomical prognosis after surgery in iERM.

## Materials and methods

### Study population

The Department of Ophthalmology and Visual Science at the Catholic University of Korea’s Seoul St. Mary’s Hospital, served as the site for this retrospective observational study. This study follows the principles outlined in the Declaration of Helsinki and received approval from the Institutional Review Board (IRB) of the Catholic University of Korea for all protocols (KC23RASI0092). In adherence to the IRB guidelines, written informed consent procedures were waived for this study due to its retrospective nature and the anonymization of data. Data between September 27th, 2021 and February 28th, 2023 were accessed for this study from February 17th, 2023 to February 16th, 2024 in accordance with IRB approval.

From September 2021 to August 2022, a total of 28 patients diagnosed with iERM and experiencing decreased visual acuity along with metamorphopsia, as detected by Amsler grid findings, underwent pars plana vitrectomy (PPV) with ILM peeling at our clinic.

Patients with a history of ocular trauma, intravitreal injection, or previous retinal surgery were excluded from the study. Additionally, cases involving ocular inflammatory disease, retinal vessel occlusion, diabetic retinopathy, or any other vitreoretinal and anterior segment diseases were excluded to ensure the reliability of the data. Those with prior optic neuropathy, glaucoma suspect, ocular hypertension, glaucoma, or significant media opacity were also excluded. We excluded patients with severely compromised visual acuity whose decimal BCVA was under 0.05.

### Study protocol

At the initial visit, demographics, medical and ophthalmologic histories were gathered from all participants. Slit-lamp microscopy and dilated fundus examination were conducted on the participants. Snellen BCVA was achieved, and presence or absence of patients’ metamorphosia was confirmed by Amsler grid. Before the surgery, the axial length was measured by the IOL-Master 700 (Carl Zeiss Meditec, Jena, Germany), and OCT/OCTA imaging was conducted using the DRI Triton SS-OCT (Topcon, Tokyo, Japan). At postoperative follow-ups, BCVA measurement and OCT were performed at least after 6 months from the operation day. According to the previous study, postoperative BCVA and OCT results showed a trend of improvement even at 6 months after the operation [21].

Each OCT/OCTA image was thoroughly examined and analyzed independently. J.L. who was blinded to the clinical and functional status of the subjects extracted OCT/OCTA images and removed the personal information from those images. Two retinal specialists, Y-H.P. and G-H.K., were given those censored images and manually measured CFT and CCT. They were blinded to the measurements of each other, and the measurements were averaged. Considering the potential influence of patient demographics on the vasculature of retina and choriocapillaris, we evaluated the unaffected fellow eyes of all subjects as controls.

### Surgical technique

The experienced surgeon (Y-H.P.) performed a standard 25-gauge 3-port PPV using the Constellation device (Alcon, Fort Worth, TX, USA) under general anesthesia. If required, the surgeon performed phacoemulsification and intraocular lens implantation (Artis PL E; Cristalens Industrie, Lannion, France) in the posterior chamber prior to the vitrectomy. In each patient, a complete vitrectomy was conducted, ensuring the elevation and trimming of the posterior hyaloid membrane until reaching the vitreous base straddling the ora serrata. The ERM was carefully peeled off, along with the ILM, using forceps within an area of at least 2-disc diameter surrounding the fovea after applying 0.05 mL of 0.05% indocyanine green (Diagno green; Cheil Pharm. Co., Korea) on the macula for one minute. Periphery of the retina was inspected thoroughly, argon laser barrier photocoagulation was carried out around any degenerative lesions, or retinal breaks identified during the inspection.

### OCT/OCTA image analysis

OCT/OCTA images were obtained for both eyes the day prior to the surgery for preoperative evaluation. Postoperative OCT images were captured for the eyes with ERM more than 6 months after the surgery. The image quality scores of all OCT scans were above 65 points and OCTA scans above 50 points. Images with motion artifacts and/or stretch artifacts were also excluded. Mean image quality of OCT was 89.07, and OCTA was 64.43 in this study.

**Fig 1:**
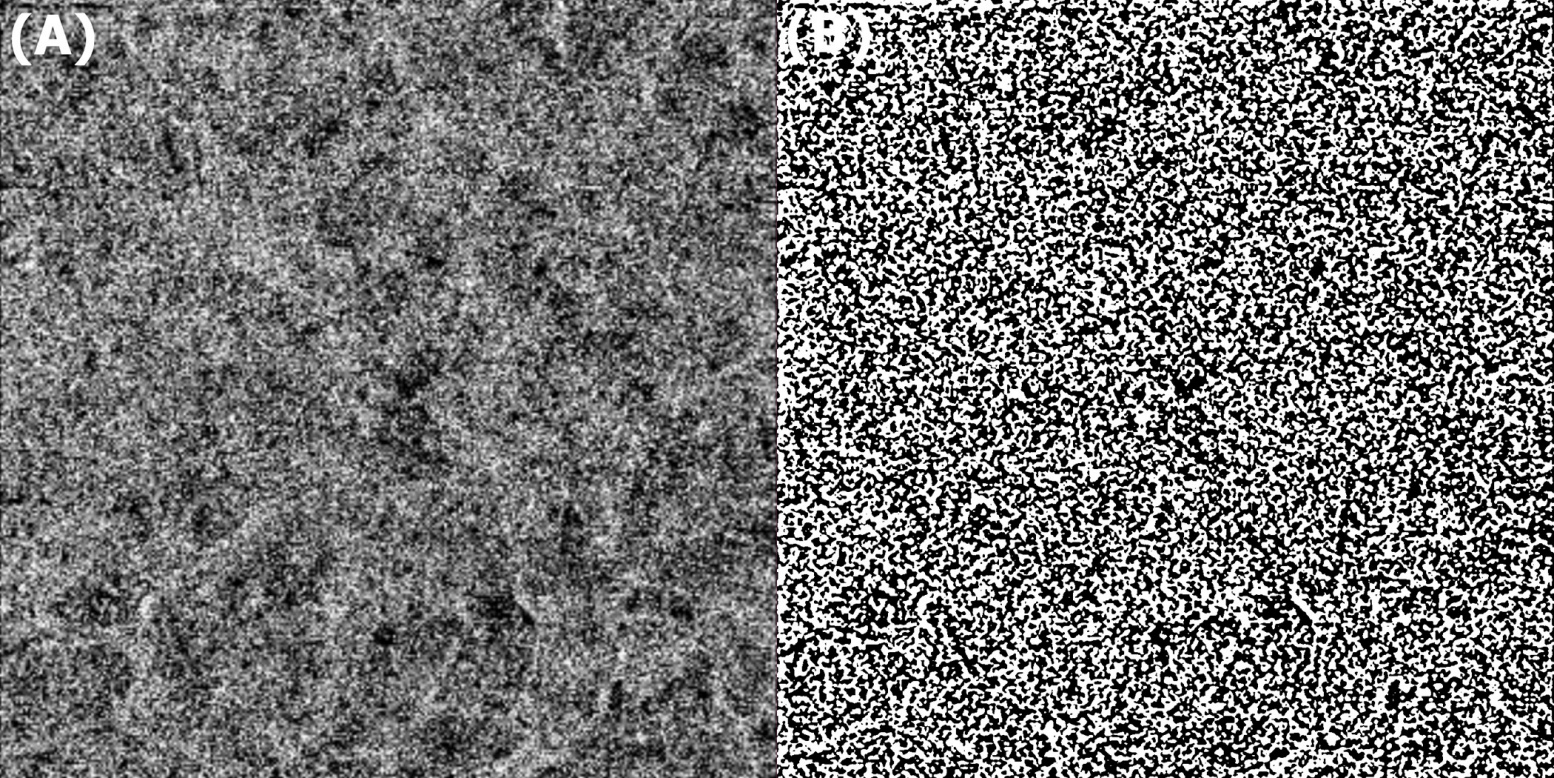
Vascular density measurement with optical coherence tomography angiography (OCTA). (A) The en-face OCTA image of choriocapillaris was obtained from Topcon image-NET 6 software. (B) The image was binarized by Otsu’s autolocal threshold to demarcate the luminal and stromal area for measuring vascular densities by Image J software.

**Fig 2:**
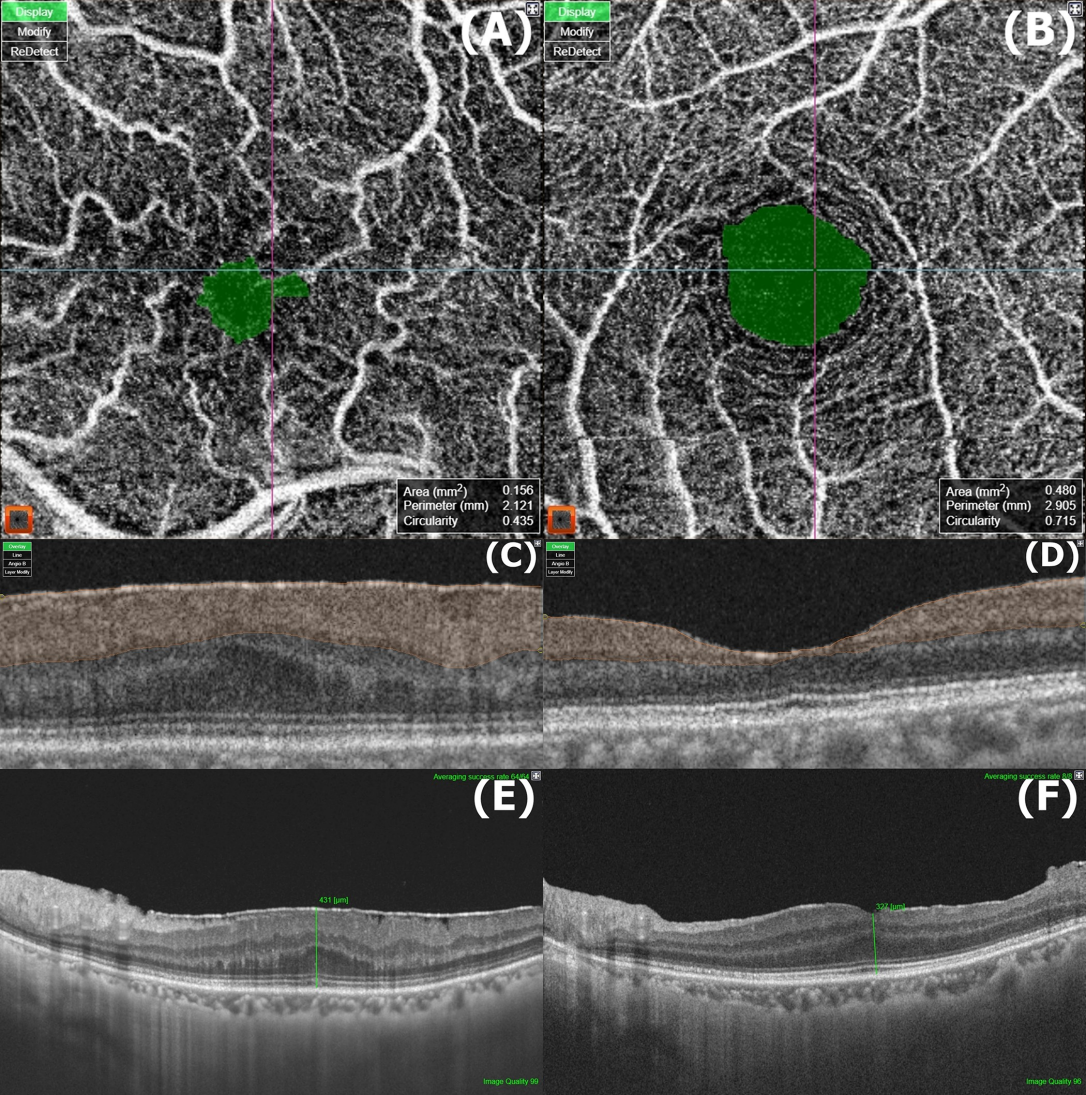
Representative optical coherence tomography angiography (OCTA) images of preoperative superficial foveal avascular zone (FAZ). The en-face FAZ analysis (green area on images) of (A) idiopathic epiretinal membrane (iERM) eye (area = 0.156 mm^2^, perimeter = 2.121 mm, circularity = 0.435) and (B) unaffected fellow eye (area = 0.480 mm^2^, perimeter = 2.905 mm, circularity = 0.715) of the same patient. Superficial FAZ measurement ranges were correspondingly shown in OCTA B-scan images, (C) and (D) (orange zone, from internal limiting membrane to the inner plexiform layer). OCT B-scan images of the same iERM eye. (E) Preoperative Central foveal thickness (CFT) = 431 μm, (F) postoperative CFT = 327 μm.

We obtained the en-face choriocapillaris images from the 4.5 × 4.5 mm OCTA scans automatically segmented by Topcon image-NET 6 software. The obtained images contained 16-bit greyscale data for each pixel. Due to the requirements of Image J software, each pixel of the images was converted into 8-bit greyscale by linear mapping before further processing. After then, to distinguish which part of the image corresponds to the capillaries or not, we created a binary image segmenting the 8-bit grayscale image into a particle region (luminal area) and a background region (stromal area) based on threshold values provided by Otsu’s local adaptive algorithm. These modified images were finally analyzed by the ‘Vascular Density (VD)’ command in Image J software (version 1.53a; National Institute of Health, USA) to achieve CCP, as suggested by Nicolò et al. (Fig 1) [29]. The superficial FAZ was analyzed OCTA scans between the ILM and the inner plexiform layer by Topcon image-NET 6 software, which simultaneously provides area (FAZa), perimeter (FAZp), and circularity (FAZc) of the FAZ (Fig 2(A)–2(D)).

We conducted a morphological analysis of the macula using SS-OCT images in the 12 mm x 12 mm 3D scan mode. ERMs were divided into 4 stages according to the recently suggested OCT-based staging system [13]. The central foveal thickness (CFT) before and after the surgery was selected as a representative parameter of the retinal morphology. CFT ratio was defined as ratio of post- to preoperative CFT. Similarly, we measured the central choroidal thickness (CCT) to evaluate morphological characteristics of the choroid. Two experienced retinal specialists independently assessed it using the digital caliper tool available in Topcon image-NET 6 software (Fig 2(E) and 2(F)), and the measurements were averaged. Inter-observer differences were assessed by using the intra-class correlation (ICC) for the manual measurements.

### Statistical analysis

The continuous variables were reported as the mean ± standard deviation (SD). We assessed the normal distribution of the data before selecting the appropriate statistical analysis methods. To assess the changes in best-corrected visual acuity (BCVA) following the operation, the BCVA measurements were transformed into the logarithm of the minimum angle of resolution (LogMAR). Functional and anatomical improvements following the surgery was analyzed by the Wilcoxon signed-rank test. Bivariate Pearson correlation tests were performed to show correlations of preoperative parameters with functional and anatomical outcomes for exploratory purposes. Multiplicity corrections were not performed in the search for potential relationships. Ultimately, stepwise multivariate regression analyses including previously suggested predictors were performed to reveal significant biomarkers of ERM surgery. Variables with a variance inflation factor of more than 5 were considered to have excessive collinearity and were excluded. The statistical analysis was conducted using the Statistical Package for the Social Sciences for Windows (Version 26.0, SPSS Inc., Chicago, IL). Statistical significance would be established when *P*-value is under 0.05.

## Results

This study included data from 28 eyes of 28 patients, comprising 5 males and 23 females, with ages ranging from 55 to 77 years (mean age 64.0 ± 6.6 years). Out of the total, only one eye (3.6%) was pseudophakic, while the remaining eyes (96.4%) had clear crystalline lenses and underwent phacovitrectomy. There were 13 eyes at stage 2, 10 at stage 3, five at stage 4, and none at stage 1. Demographic data and preoperative ocular characteristics of the subjects are provided in Table 1.

**Table 1:** Demographics of the patients and characteristics of the study eyes.

Total Number of Eyes	28
Age (years)	64.0 ± 6.6
Axial length (mm)	23.6 ± 1.3
Laterality (right: left)	11: 17
Sex (male: female)	5: 23
Lens status (phakia: pseudophakia)	27: 1
Stage (1: 2: 3: 4)	0: 13: 10: 5

Data are presented as the mean ± standard deviation or a ratio, as appropriate.

Before surgery, FAZ was shrunk (FAZa, *p* < 0.001; FAZp, *p* < 0.001) and distorted (FAZc, *p* = 0.003), CCP was reduced (*p* < 0.001), and CCT was rather increased (*p* < 0.001) significantly compared with the unaffected fellow eyes as shown in Table 2.

**Table 2:** Preoperative FAZ parameters, CCP and CCT.

	ERM eyes	Fellow eyes	Ratio	*p*
FAZa (mm^2^)	0.144 ± 0.114	0.355 ± 0.081	0.415 ± 0.320	<0.001
FAZp (mm)	1.714 ± 0.662	2.662 ± 0.366	0.653 ± 0.265	<0.001
FAZc	0.540 ± 0.101	0.623 ± 0.060	0.879 ± 0.203	0.003
CCP (%)	47.581 ± 0.666	48.300 ± 0.678	0.985 ± 0.017	<0.001
CFT (μm)	524.4 ± 95.3	406.7 ± 67.4	0.779 ± 0.173	<0.001
CCT (μm)	248.5 ± 67.6	222.4 ± 65.8	1.137 ± 0.170	<0.001

Data are presented as the mean ± standard deviation.

ERM = Epiretinal Membrane, FAZ = Foveal Avascular Zone, FAZa = FAZ area, FAZp = FAZ perimeter, FAZc = FAZ circularity, CCP = Choriocapillaris Perfusion, CFT = Central Foveal Thickness, CCT = Central Choroidal Thickness.

LogMAR BCVA was 0.543 ± 0.223 (0.2–1.2) before surgery, and it became 0.318 ± 0.174 (0–0.7) after surgery. Significant improvement of BCVA by ERM surgery was observed (*p* = 0.001). CFT was 524.4 ± 95.3 μm (370–804 μm) before surgery, and 406.7 ± 67.4 μm (197–534 μm) after surgery. CFT was significantly reduced by operation (*p* < 0.001). CCT was 248.5 ± 67.6 μm (128–379 μm) before surgery, and 222.4 ± 65.8 μm (114–368 μm) after surgery. CCT also exhibited a notable thinning after surgical intervention in this study (*p* < 0.001). ICC for the manual measurements of CFT and CCT by two researchers (Y-H.P. and G-H.K.) was 0.984 (95% CI 0.966–0.992) and 0.989 (95% CI 0.972–0.995) for preoperative evaluations, and 0.987 (95% CI 0.976–0.995) and 0.991 (95% CI 0.982–0.997) for postoperative evaluations.

**Fig 3:**
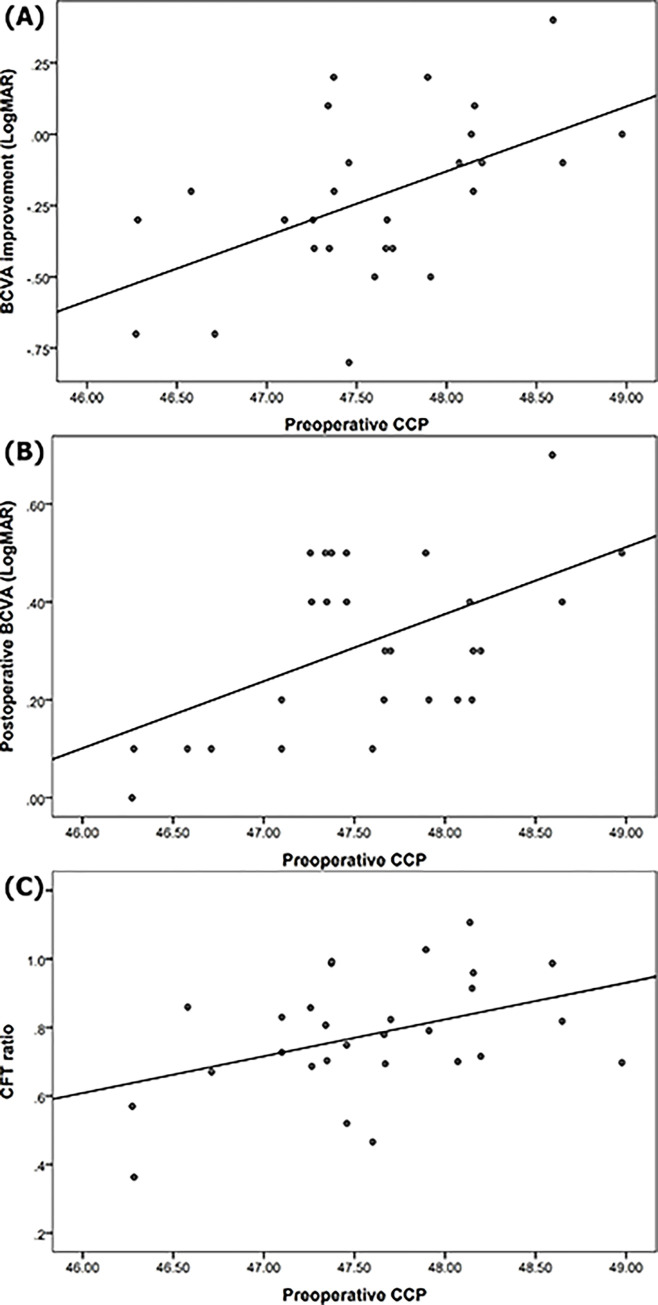
Scatterplots to exhibit correlations between preoperative choriocapillaris perfusion (CCP) versus (A) best-corrected visual acuity (BCVA) improvement (LogMAR, Logarithm of the minimum angle of resolution) (R^2^ = 0.280, *p* = 0.004), (B) postoperative BCVA (LogMAR) (R^2^ = 0.274, *p* = 0.004), and (C) central foveal thickness (CFT) ratio (= Postoperative CFT / Preoperative CFT) (R^2^ = 0.171, *p* = 0.029).

Relationship between OCTA findings and functional prognosis of iERM is described in Table 3. Preoperative FAZa showed significant correlation with preoperative BCVA (*p* = 0.003) and BCVA improvement (*p* < 0.001). Preoperative FAZc was significantly associated with postoperative BCVA (*p* = 0.002). Preoperative CCP had significant correlations with preoperative FAZc (*p* = 0.023), BCVA improvement (*p* = 0.004) (Fig 3(A)), and postoperative BCVA (*p* = 0.004) (Fig 3(B)).

**Table 3:** Correlations of visual acuity with preoperative parameters.

Preop	Preop FAZa		Preop FAZp		Preop FAZc		Postop BCVA		BCVA improvement	
	r	*p*	r	*p*	r	*p*	r	*p*	r	*p*
BCVA	**-0.535**	**0.003**	**-0.571**	**0.001**	-0.152	0.440	-0.020	0.918	**-0.793**	**<0.001**
FAZa	-	-	**0.947**	**<0.001**	0.141	0.475	0.011	0.956	**0.424**	**0.025**
FAZp	**0.947**	**<0.001**	-	-	-0.083	0.675	0.186	0.342	**0.569**	**0.002**
FAZc	0.141	0.475	-0.083	0.675	-	-	**-0.570**	**0.002**	-0.229	0.241
CFT	-0.389	0.041	-0.499	0.007	-0.058	0.770	-0.252	0.196	**-0.682**	**<0.001**
CCP	-0.187	0.340	-0.049	0.806	**-0.429**	**0.023**	**0.523**	**0.004**	**0.529**	**0.004**
CCT	-0.077	0.695	-0.176	0.372	0.204	0.297	0.102	0.604	-0.059	0.767
AxL	-0.140	0.479	-0.091	0.643	-0.002	0.994	-0.028	0.887	-0.074	0.708

Statistically significant *P*-value is shown in bold.

(Multiplicity corrections were not performed due to the exploratory purpose.)

Preop = Preoperative, Postop = Postoperative, BCVA = Best-Corrected Visual Acuity (LogMAR = Logarithm of the minimum angle of resolution), FAZ = Foveal Avascular Zone, FAZa = FAZ area, FAZp = FAZ perimeter, FAZc = FAZ circularity, CFT = Central Foveal Thickness, CCP = Choriocapillaris perfusion, CCT = Central Choroidal Thickness, AxL = Axial Length.

BCVA improvement = Postoperative BCVA (LogMAR)—Preoperative BCVA (LogMAR).

r, Correlation Coefficient; *p* = Significant Value.

We also investigated correlations between OCTA findings and anatomical prognosis of iERM. Preoperative CCP showed significant association with the morphological improvement after ERM surgery as CFT ratio (*p* = 0.029) (Fig 3(C)). Preoperative CCT was highly correlated with postoperative CCT (*p* < 0.001), and they showed significant inverse associations with the axial length (Preoperative CCT, *p* = 0.011; Postoperative CCT, *p* = 0.007) (Table 4).

**Table 4:** Correlations of retinal morphology with preoperative parameters.

Preop	Preop CFT		Postop CFT		CFT ratio		Preop CCT		Postop CCT	
	r	*p*	r	*p*	r	*p*	r	*p*	r	*p*
BCVA	**0.677**	**<0.001**	0.007	0.972	**-0.436**	**0.021**	0.145	0.461	0.119	0.548
FAZa	**-0.389**	**0.041**	0.004	0.984	0.273	0.160	-0.082	0.679	-0.052	0.793
FAZp	**-0.499**	**0.007**	0.101	0.611	**0.415**	**0.028**	-0.186	0.342	-0.173	0.379
FAZc	-0.058	0.770	-0.299	0.122	-0.195	0.319	0.216	0.269	0.255	0.190
CCP	-0.252	0.196	0.299	0.123	**0.414**	**0.029**	0.092	0.640	0.085	0.668
CFT	**-**	**-**	0.011	0.954	**-0.676**	**<0.001**	-0.033	0.869	-0.041	0.837
CCT	-0.033	0.869	0.264	0.174	0.266	0.172	-	-	**0.989**	**<0.001**
AxL	0.106	0.590	-0.122	0.535	-0.197	0.316	**-0.476**	**0.011**	**-0.497**	**0.007**

Statistically significant *P*-value is shown in bold.

(Multiplicity corrections were not performed due to the exploratory purpose.)

Preop = Preoperative, Postop = Postoperative, BCVA = Best-Corrected Visual Acuity (LogMAR = Logarithm of the minimum angle of resolution), FAZ = Foveal Avascular Zone, FAZa = FAZ area, FAZp = FAZ perimeter, FAZc = FAZ circularity, CCP = Choriocapillaris perfusion, CFT = Central Foveal Thickness, CCT = Central Choroidal Thickness, AxL = Axial Length.

CFT ratio = Postoperative CFT / Preoperative CFT.

r, Correlation Coefficient; *p*, Significant Value.

Since multi-collinearities were not shown, all potential prognostic variables were adjusted for multivariate analysis. Multivariate regression analysis revealed that preoperative CCP was significantly related with BCVA improvement (β = 0.431, *p* = 0.005), postoperative BCVA (β = 0.412, *p* = 0.048) and CFT ratio (β = 0.407, *p* = 0.044) (Table 5). Preoperative FAZc showed negative relationship with postoperative BCVA (β = -0.386, *p* = 0.038). Preoperative FAZa (β = 0.348, *p* = 0.013) and CFT (β = -0.445, *p* = 0.003) had correlations with BCVA improvement.

**Table 5:** Multiple linear regression analysis for functional and anatomical outcomes.

Preoperative	BCVA improvement (R^2^ = 0.706)		Postoperative BCVA (R^2^ = 0.517)		CFT ratio (R^2^ = 0.330)	
	β	*p*	β	*p*	β	*p*
BCVA	-	-	0.377	0.133	-0.195	0.389
FAZa	**0.348**	**0.013**	0.210	0.294	0.245	0.273
FAZc	-0.119	0.361	**-0.386**	**0.038**	-0.118	0.564
CCP	**0.431**	**0.005**	**0.412**	**0.048**	**0.407**	**0.044**
CFT	**-0.445**	**0.003**	-0.344	0.105	-	-

Statistically significant *P*-value is shown in bold.

BCVA = Best-Corrected Visual Acuity (LogMAR = Logarithm of the minimum angle of resolution), FAZ = Foveal Avascular Zone, FAZa = FAZ area, FAZc = FAZ circularity, CCP = Choriocapillaris Perfusion, CFT = Central Foveal Thickness.

CFT ratio = Postoperative CFT / Preoperative CFT.

β, Standard Regression Coefficient; *p*, Significant Value.

## Discussion

Using OCTA, we conducted a study to explore vascular changes in the retina and choroid of eyes with iERM. Additionally, we performed multivariate regression analyses utilizing retinal and choriocapillaris parameters to identify potential biomarkers that could predict postoperative outcomes. We believe that our study is the first to support the hypothesis that preoperative CCP could be a biomarker for poor postoperative BCVA and CFT reduction following surgical intervention.

There is no definite consensus on the indications for surgical intervention in iERM [30]. The selection of surgical patients is based on various factors, including the symptoms experienced by patients, their visual needs, and the technical expertise of the surgeon. However, patients with iERM typically exhibit moderately good preoperative vision [30]. As a result, the selection process should be conducted with caution, and there has been an increased emphasis on the significance of preoperative predictors for postoperative visual outcomes.

It is easy to observe an increase in vascular tortuosity and the contraction of the retina in patients with iERM through fundus oculi examination, but quantifying these changes proves to be challenging. OCTA has enabled the assessment of various parameters that can be utilized in evaluating the extent of distortion and the impact of contractions on the retina with iERM. As previously reported, this study found that the preoperative FAZ area was generally shrunk and distorted in eyes with iERM compared to their unaffected fellow eyes [1, 31, 32]. Also, greater level of preoperative FAZ contraction was correlated with lower preoperative visual acuity and more significant improvement in visual acuity by ERM surgery same as previous studies [1, 31, 32]. There appears to be a relationship between a more contracted FAZ and increased mechanical stress on the outer retina, and it is known that the FAZ tends to enlarge after ERM surgery [1, 32]. When the ERM is solely linked to mechanical stress without causing fragmentation of the ellipsoid zone, surgical removal of the ERM has the potential to enhance visual acuity in proportion to the extent of mechanical stress exerted by the ERM. However, final visual function in ERM eyes seems to be limited by residual retinal deformity which is hard to be improved by conventional ERM peeling. This study found that postoperative BCVA was significantly correlated with preoperative FAZc, which is hard to change, unlike FAZa or FAZp, after surgical intervention [21].

The inner-retinal layer alterations have received significant interest in eyes with ERM, as ERM primarily affects the inner-retinal structure. However, in the era of EDI-OCT, the accurate assessment of choroidal involvement in ERM has been significantly improved. Previous studies have demonstrated that the impact of ERM on the choroid is dependent on the level of tractional force exerted [15]. It was postulated that the traction-induced alterations in retinal blood flow might stimulate the choroidal vessels to compensate for the elevated oxygen and nutrient demands [15]. However, others observed that there was no notable alteration in CVI values as the stages of ERM and levels of contraction increased [15]. Recently, one study analyzed changes of CVI in Sattler’s and Haller’s layers separately, and reported meaningful change only in Sattler’s layer, which is nearer to retina than Haller’s layer [19].

Previous studies reported a decrease in CCP in eyes with iERM compared to their unaffected fellow eyes or normal eyes, which was found to be reversible following surgery [19, 20]. Given consistent findings about CCP changes from previous literature, we focused on choriocapillaris which is not directly affected by ERM, but closely influenced as the nearest layer. In this study, we found that preoperative CCP is not only significantly reduced in iERM eyes than unaffected fellow eyes, but also correlated with worse preoperative FAZc, postoperative BCVA, and CFT.

Several potential mechanisms have been proposed to elucidate alterations in the choroid and choriocapillaris in ERM pre- and post-surgery, such as anteroposterior forces, local change of vascular endothelial growth factor (VEGF) level, and vitreous oxygenation after vitrectomy [19]. Moreover, irreversible disruption of retinal layer due to ERM contraction may lead to irreversible compensation for the increased oxygen and nutrient needs [19]. Above potential mechanisms could possibly explain the significant correlations of preoperative CCP with worse functional and anatomical prognosis in this study. However, it is still necessary to explain why Sattler’s layer of choroid and choriocapillaris seem to change differently in ERM before and after surgery. We suggest that the difference might be caused by the current measurement method. First, choriocapillaris consists of far smaller vessels than choroid, so it would be difficult to capture vasculature of choriocapillaris as fully as choroid [33]. Second, CCP has been analyzed from SS-OCTA scans, but CVI from SS-OCT scans. According to previous study, SS-OCTA has underestimated inner choroidal vasculature compared to SS-OCT [33]. ERM which needs surgery is usually a thick hyper-reflective layer over inner surface of the retina, so it could cause additional underestimation of choriocapillaris vasculature by OCTA.

To sum up, the seemingly contradictory results of this study could be explained as follows. If ERM, a thick hyper-reflective layer, exists as an interfering factor, CCP is observed on OCTA as if reduced. However, as the degree of disruption of retinal layer due to ERM becomes more severe, CCP may increase according to various mechanisms suggested above.

Although some previous studies have reported correlations between pre- and postoperative BCVA, this study indicates no such correlation (r = -0.020, *p* = 0.918). On the other side, preoperative CCP and FAZc seem to predict postoperative visual function in this study. To explain this discrepancy, we would like to suggest that the deterioration of visual function caused by ERM should be viewed separately into reversible and irreversible parts. FAZa is known to be well improved by ERM peeling, and there have been many past reports about inverse relationship between preoperative FAZa and the degree of BCVA improvement after surgery. Therefore, preoperative FAZa is thought to be closely related to the reversible part. However, FAZc cannot be improved well even with surgery, so it might be closely related to irreversible part. In cases of severe ERM which necessarily need surgical interventions, FAZc appears to act as a limiting factor in prognosis of visual function and may show significance with postoperative BCVA as in this study. Moreover, preoperative CCP showed significant correlation with preoperative FAZc and postoperative BCVA, so it also appears to reflect irreversible vision deterioration. Previously suggested mechanisms for the relationship between CCP and irreversible disruption of the retina can support this explanation in a similar context.

This study has some limitations. The first limitation is the retrospective nature of the study, which includes a relatively small number of subjects. Secondly, errors in the segmentation process may arise due to ERM contraction, potentially resulting in inaccuracies in vasculature analysis. While our analysis using the built-in automatic tool of Topcon image-NET 6 software helped mitigate human bias, it is important to acknowledge that inadvertent technical errors could still be present. The third one is that postoperative OCTA was not performed in our study due to economic constraints. Although previous studies have consistently reported results comparing pre- and postoperative OCTA scans in ERM [19, 20], it would have been beneficial to independently verify these findings in our study. We hope that future research will prospectively confirm the significant findings of our study.

## Supporting information

S1 Table Raw data for replicating this study findings. (PDF)

S1 File Zip file containing en-face OCTA choriocapillaris images of enrolled eyes. (ZIP)
