# Supplementary material for: Exploratory analysis of choriocapillaris vasculature as a biomarker of idiopathic epiretinal membrane
Source: PLoS One. 2024 Jul 5;19(7):e0306735. doi: 10.1371/journal.pone.0306735 (PMC11226130; doi:10.1371/journal.pone.0306735)
Supplement: S1 Table — (PDF) [file pone.0306735.s001.pdf]

					Study eye														Fellow eye				
Pt. No.	Operation date	Gender	Age	ERM laterality	FAZa	FAZp	FAZc	CCP	VA_base	VA_6m	VA impr	ERM stage	CFT_base	CFT_6m	CFT ratio	CCT_base	CCT_6m	AxL	FAZa	FAZp	FAZc	CCP	CCT
1	20210928	F	62	OS	0.058	1.182	0.522	48.776	0.5	0.5	0	3	552	385	0.697464	225	215	24.8	0.141	1.763	0.568	49.015	255
2	20210928	F	54	OS	0.156	2.121	0.435	48.397	0.4	0.3	-0.1	2	531	380	0.715631	128	119	22.8	0.48	2.905	0.715	48.877	125
3	20211019	M	54	OS	0.109	1.718	0.466	47.099	0.4	0.1	-0.3	2	547	398	0.727605	184	143	25.9	0.356	2.551	0.689	48.656	170
4	20211026	F	66	OS	0.442	3.136	0.565	46.483	0.4	0.1	-0.3	2	543	197	0.362799	176	160	24.29	0.468	3.578	0.46	48.378	169
5	20211026	F	57	OD	0.22	2.215	0.563	48.156	0.2	0.3	0.1	2	443	425	0.959368	334	312	22.93	0.372	2.842	0.579	48.539	300
6	20211109	F	64	OS	0.019	0.621	0.626	47.6	0.6	0.1	-0.5	2	698	325	0.465616	215	190	23.8	0.399	3.179	0.496	47.151	155
7	20211124	M	69	OD	0.381	2.917	0.563	47.252	0.3	0.5	0.2	2	541	534	0.987061	175	180	23.06	0.335	2.721	0.569	47.738	166
8	20211201	F	59	OS	0.061	1.172	0.559	48.551	0.7	0.2	-0.5	3	554	438	0.790614	220	193	23.6	0.349	2.825	0.549	47.911	157
9	20211216	F	64	OD	0.053	1.142	0.51	48.646	0.5	0.4	-0.1	3	534	437	0.818352	361	335	21.76	0.382	2.633	0.691	47.207	342
10	20211222	F	69	OS	0.074	1.374	0.493	47.127	0.8	0.5	-0.3	3	569	488	0.857645	370	341	23.58	0.304	2.483	0.619	49.157	335
11	20220112	F	59	OS	0.035	1.02	0.42	47.7	0.7	0.3	-0.4	2	572	471	0.823427	232	217	25.91	0.321	2.519	0.637	47.796	227
12	20220216	F	73	OD	0.173	2.005	0.541	48.237	0.4	0.4	0	2	440	487	1.106818	330	307	22.7	0.388	2.921	0.572	48.92	240
13	20220308	M	57	OD	0.192	2.11	0.543	48.349	0.4	0.2	-0.2	2	525	480	0.914286	233	224	23.27	0.331	2.588	0.621	48.59	196
14	20220315	F	59	OD	0.09	1.563	0.461	47.264	0.8	0.4	-0.4	4	651	447	0.686636	259	233	23.23	0.395	2.712	0.675	49.106	238
15	20220315	F	59	OS	0.136	2.006	0.423	47.456	0.6	0.5	-0.1	3	565	423	0.748673	216	194	24.79	0.421	2.789	0.68	48.273	189
16	20220316	F	74	OD	0.023	0.849	0.399	47.457	1.2	0.4	-0.8	4	804	418	0.5199	303	292	22.89	0.348	2.624	0.635	49.053	258
17	20220323	F	76	OS	0.179	1.911	0.616	47.341	0.4	0.5	0.1	2	435	351	0.806897	183	167	23.11	0.492	2.988	0.692	47.56	187
18	20220323	F	76	OS	0.093	1.47	0.543	47.099	0.5	0.2	-0.3	3	512	425	0.830078	224	221	23.87	0.427	3.013	0.592	47.45	201
19	20220329	F	61	OD	0.371	2.638	0.67	46.579	0.3	0.1	-0.2	2	550	473	0.86	310	300	23.32	0.423	2.809	0.674	48.55	306
20	20220406	M	57	OS	0.179	2.494	0.362	48.142	0.3	0.7	0.4	3	463	457	0.987041	140	114	27.08	0.141	1.653	0.647	48.945	121
21	20220412	M	73	OD	0.194	2.343	0.445	47.374	0.7	0.5	-0.2	2	404	401	0.992574	250	202	22.57	0.321	2.535	0.628	47.555	246
22	20220531	F	71	OS	0.073	1.093	0.767	46.711	0.8	0.1	-0.7	3	567	380	0.670194	290	272	23.6	0.359	2.585	0.676	48.072	250
23	20220621	F	67	OS	0.105	1.387	0.687	48.07	0.3	0.2	-0.1	2	484	339	0.700413	284	275	23.72	0.372	2.667	0.658	48.02	274
24	20220719	F	61	OS	0.354	2.732	0.595	47.894	0.3	0.5	0.2	3	370	380	1.027027	379	368	22.91	0.413	2.865	0.632	48.189	328
25	20220802	F	55	OD	0.068	1.048	0.778	46.273	0.7	0	-0.7	4	588	335	0.569728	202	194	27.49	0.243	2.302	0.576	46.951	116
26	20220803	F	59	OS	0.059	1.244	0.48	47.669	0.6	0.3	-0.3	3	500	347	0.694	239	234	22.59	0.292	2.429	0.622	48.912	230
27	20220809	F	56	OS	0.068	1.312	0.495	47.663	0.6	0.2	-0.4	4	458	357	0.779476	210	191	23.82	0.329	2.558	0.632	49.304	166
28	20220809	F	56	OD	0.065	1.179	0.591	47.348	0.8	0.4	-0.4	4	583	410	0.703259	287	257	22.89	0.333	2.501	0.669	47.886	280
