# Supplementary figures and images for: Exploratory analysis of choriocapillaris vasculature as a biomarker of idiopathic epiretinal membrane
Source: PLoS One. 2024 Jul 5;19(7):e0306735. doi: 10.1371/journal.pone.0306735 (PMC11226130; doi:10.1371/journal.pone.0306735)

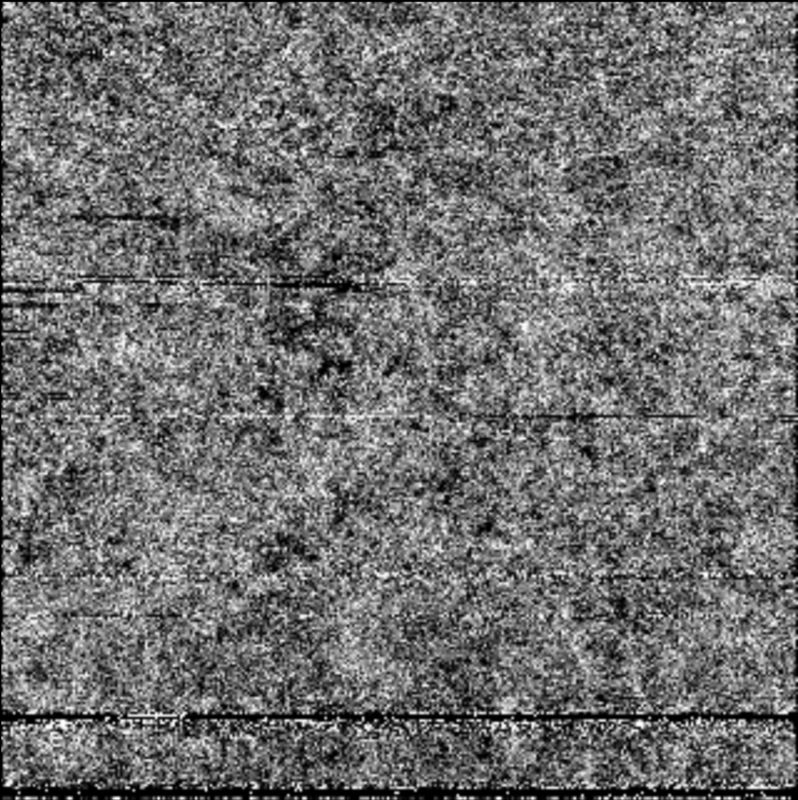

Supplement: S1 File — (ZIP) [file pone.0306735.s002.zip › 10_OD.jpg]

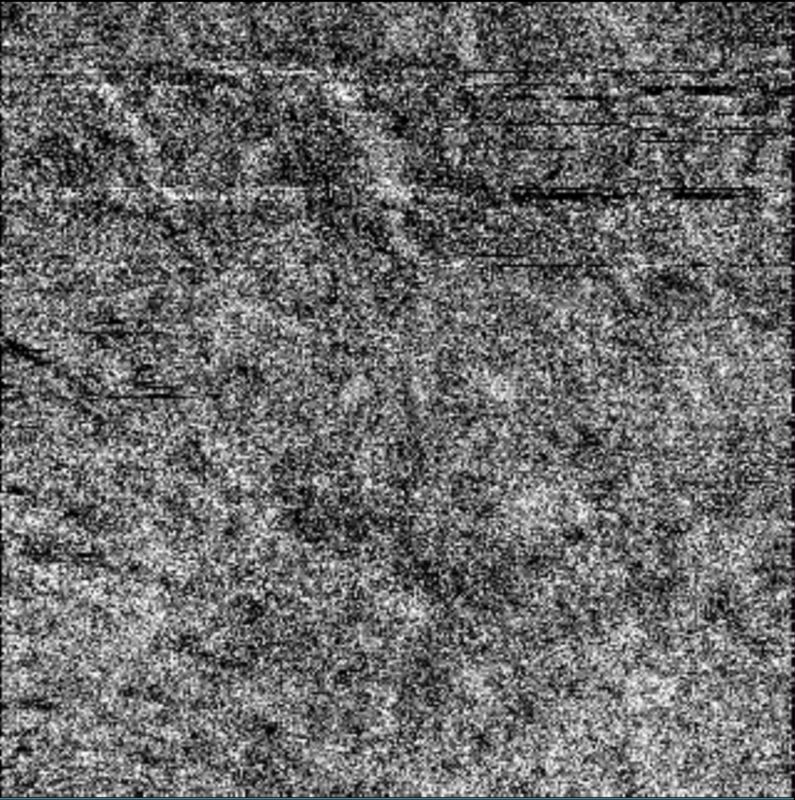

Supplement: S1 File — (ZIP) [file pone.0306735.s002.zip › 10_OS.jpg]

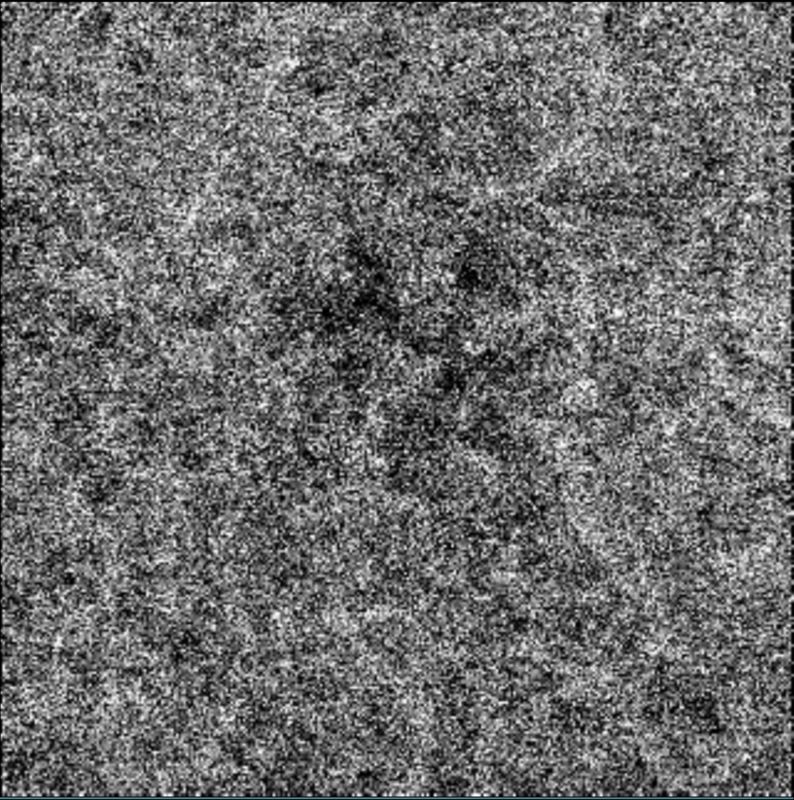

Supplement: S1 File — (ZIP) [file pone.0306735.s002.zip › 11_OD.jpg]

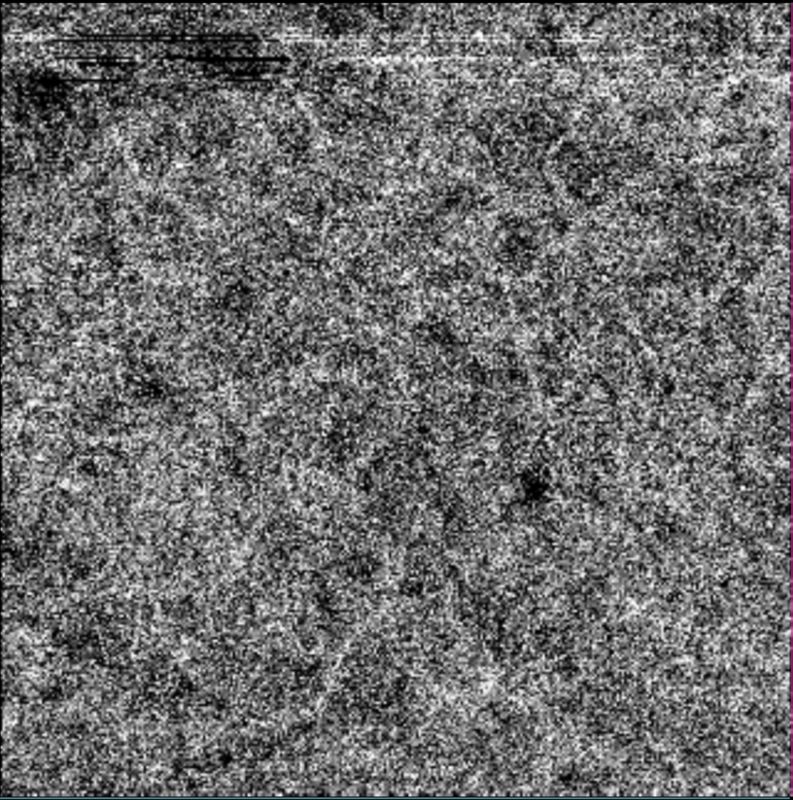

Supplement: S1 File — (ZIP) [file pone.0306735.s002.zip › 11_OS.jpg]

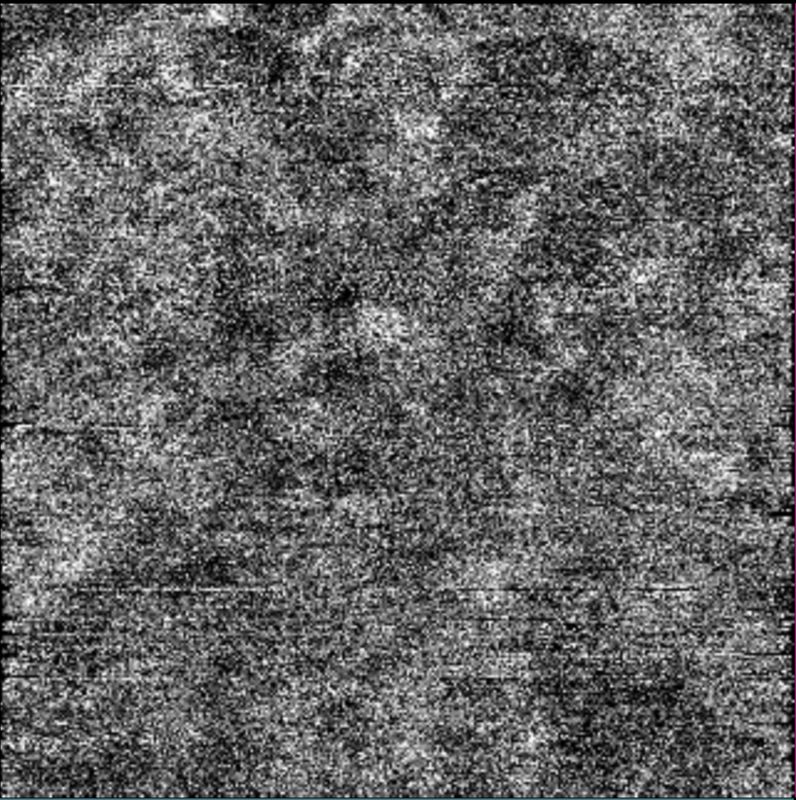

Supplement: S1 File — (ZIP) [file pone.0306735.s002.zip › 12_OD.jpg]

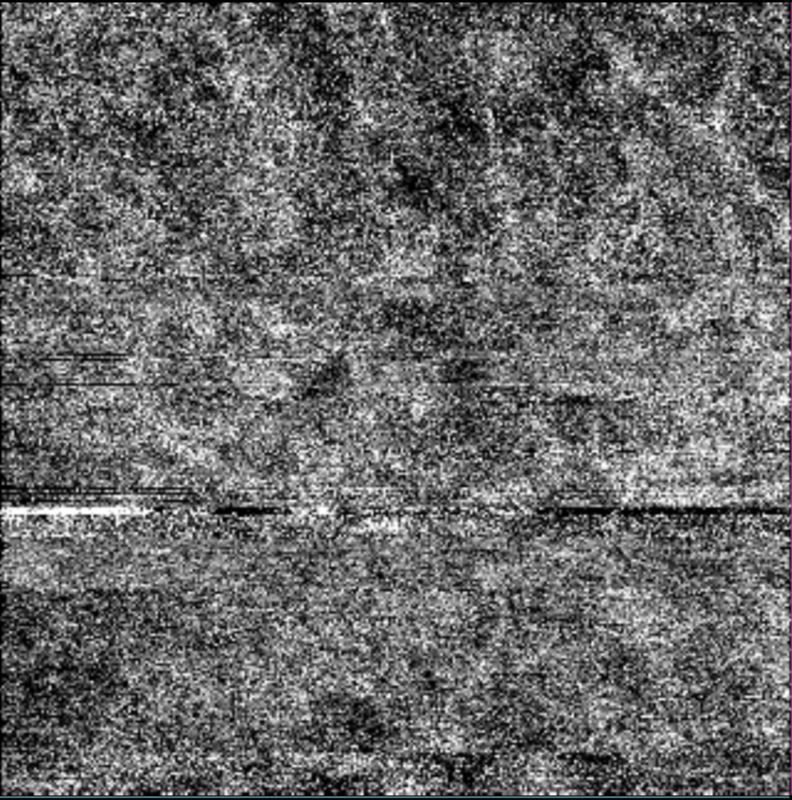

Supplement: S1 File — (ZIP) [file pone.0306735.s002.zip › 12_OS.jpg]

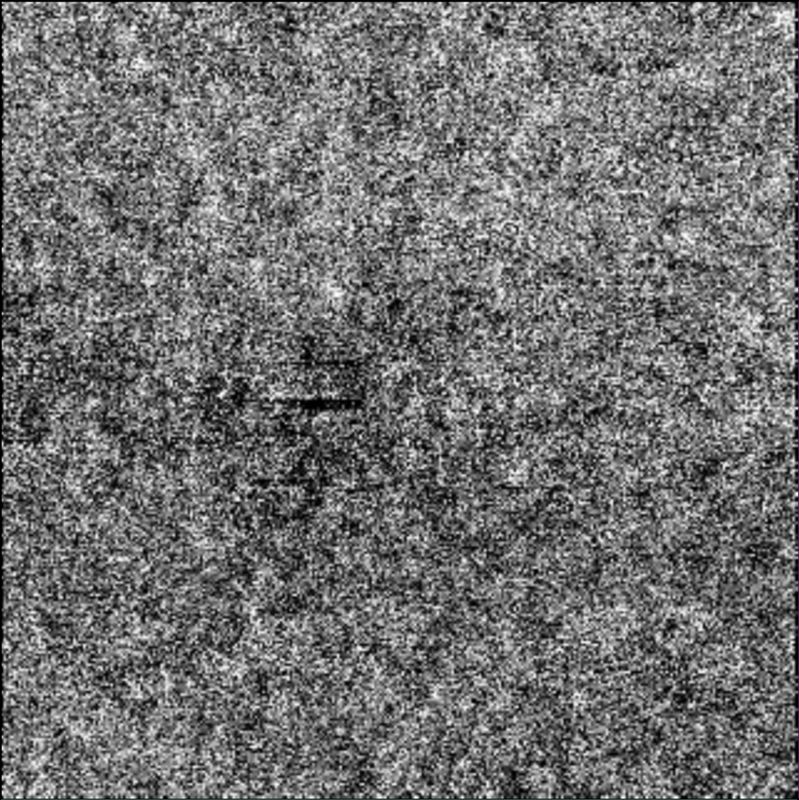

Supplement: S1 File — (ZIP) [file pone.0306735.s002.zip › 13_OD.jpg]

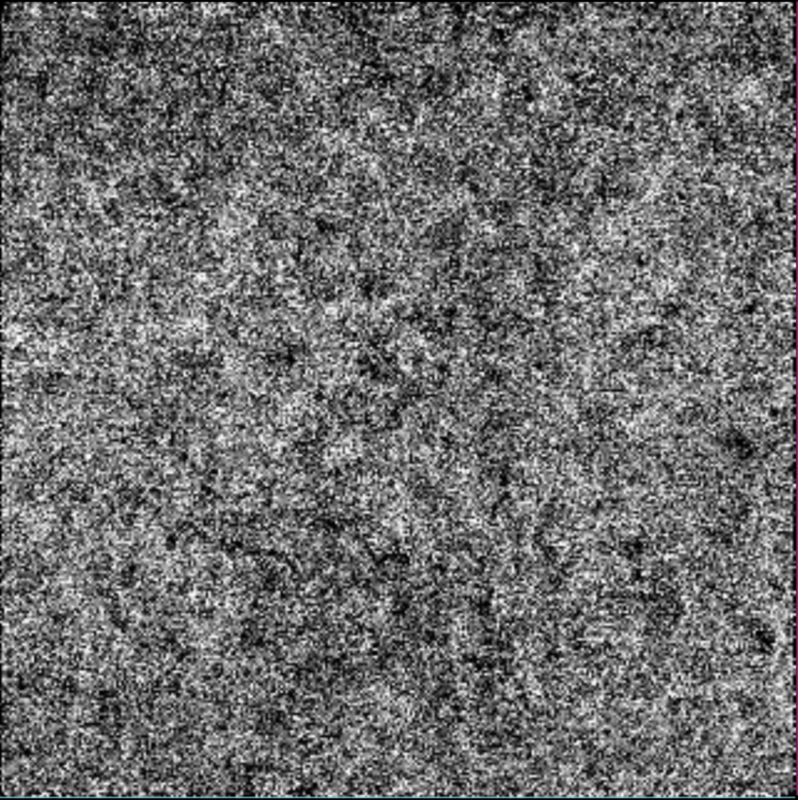

Supplement: S1 File — (ZIP) [file pone.0306735.s002.zip › 13_OS.jpg]

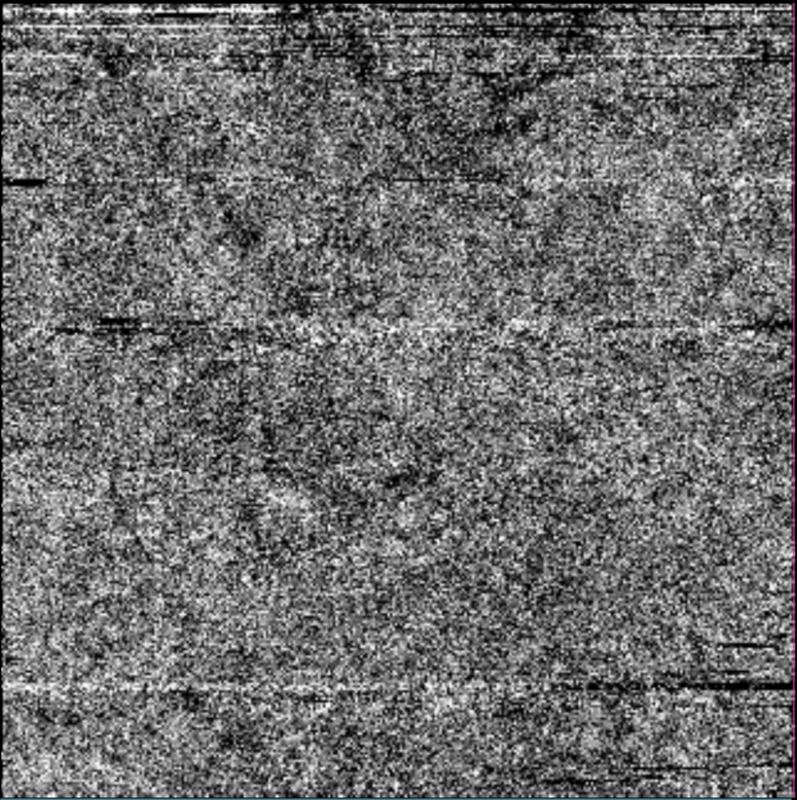

Supplement: S1 File — (ZIP) [file pone.0306735.s002.zip › 14_OD.jpg]

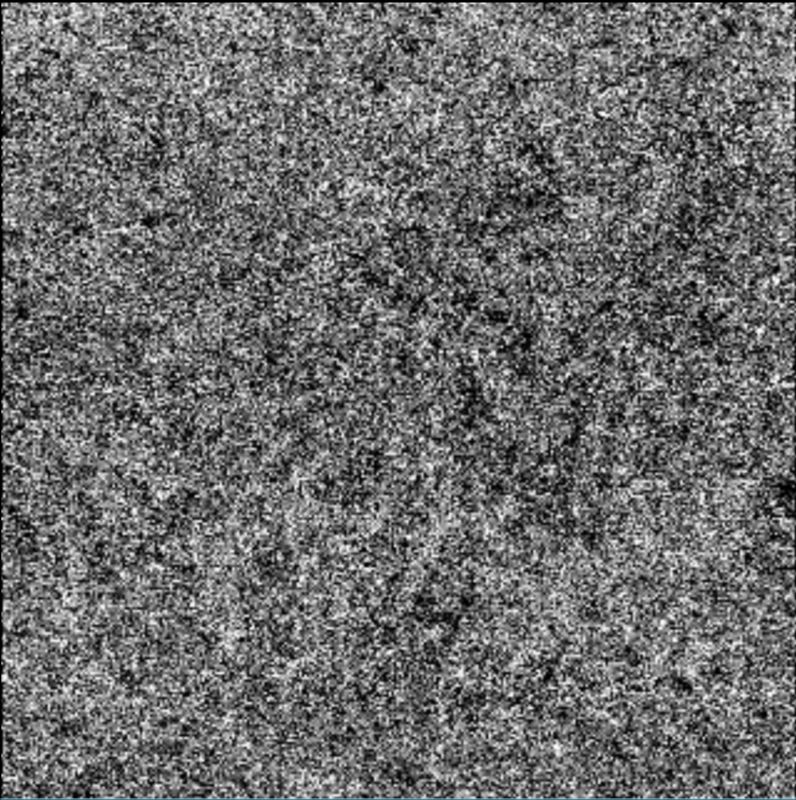

Supplement: S1 File — (ZIP) [file pone.0306735.s002.zip › 14_OS.jpg]

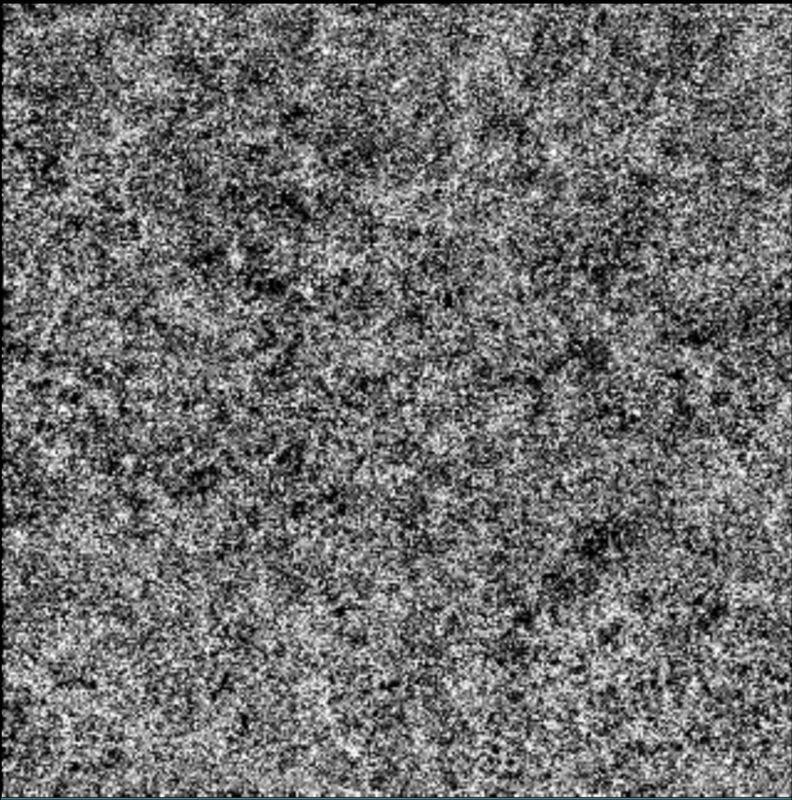

Supplement: S1 File — (ZIP) [file pone.0306735.s002.zip › 15_OD.jpg]

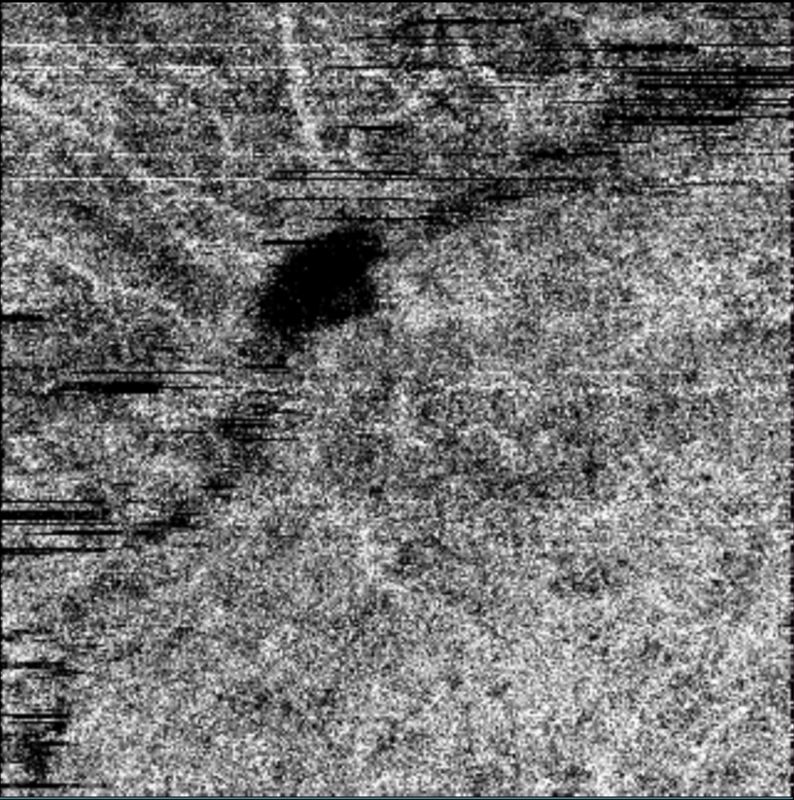

Supplement: S1 File — (ZIP) [file pone.0306735.s002.zip › 15_OS.jpg]

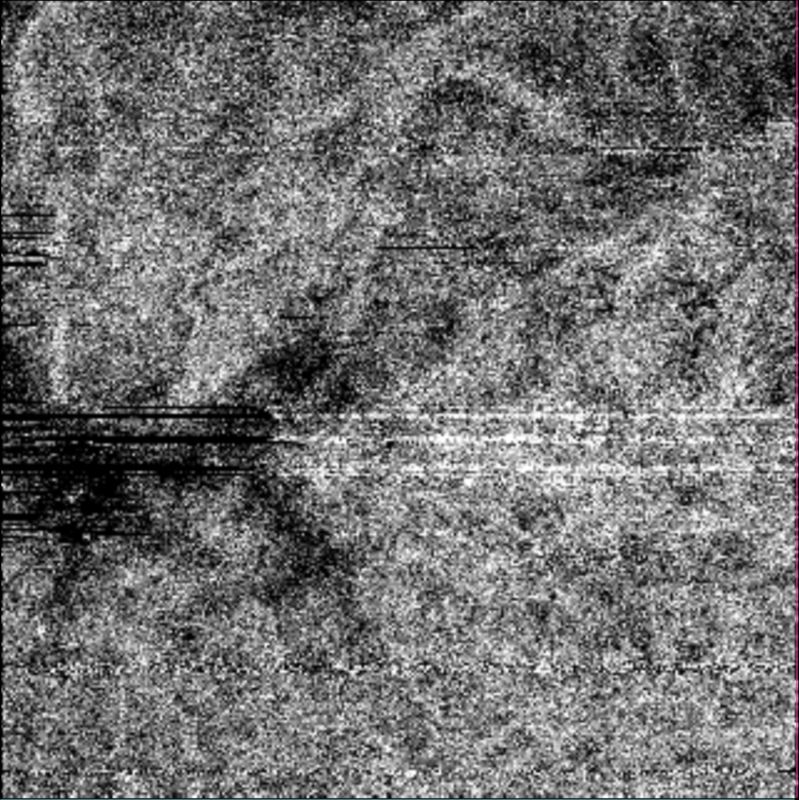

Supplement: S1 File — (ZIP) [file pone.0306735.s002.zip › 16_OD.jpg]

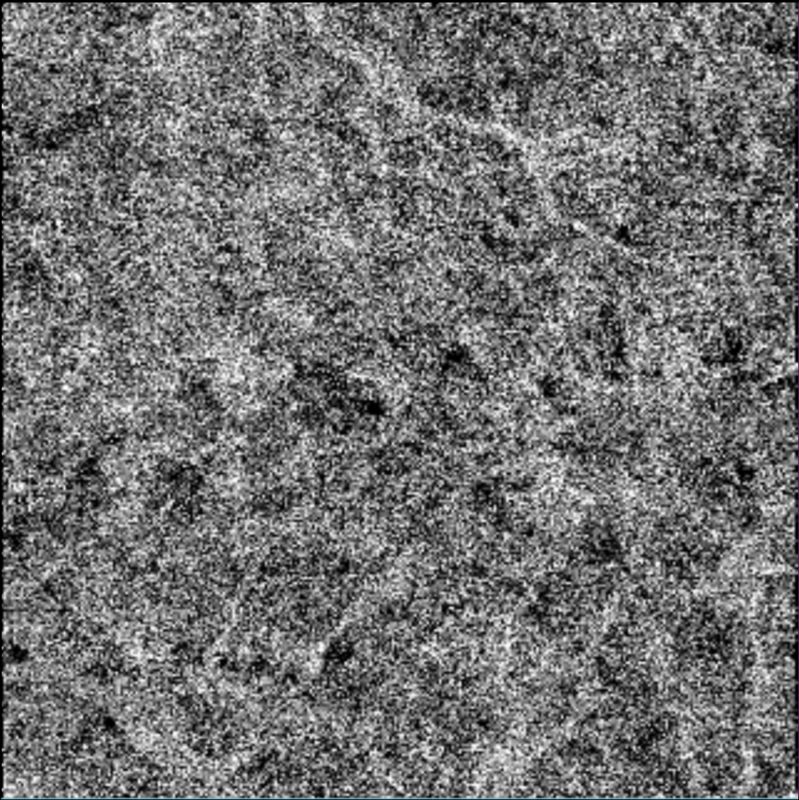

Supplement: S1 File — (ZIP) [file pone.0306735.s002.zip › 16_OS.jpg]

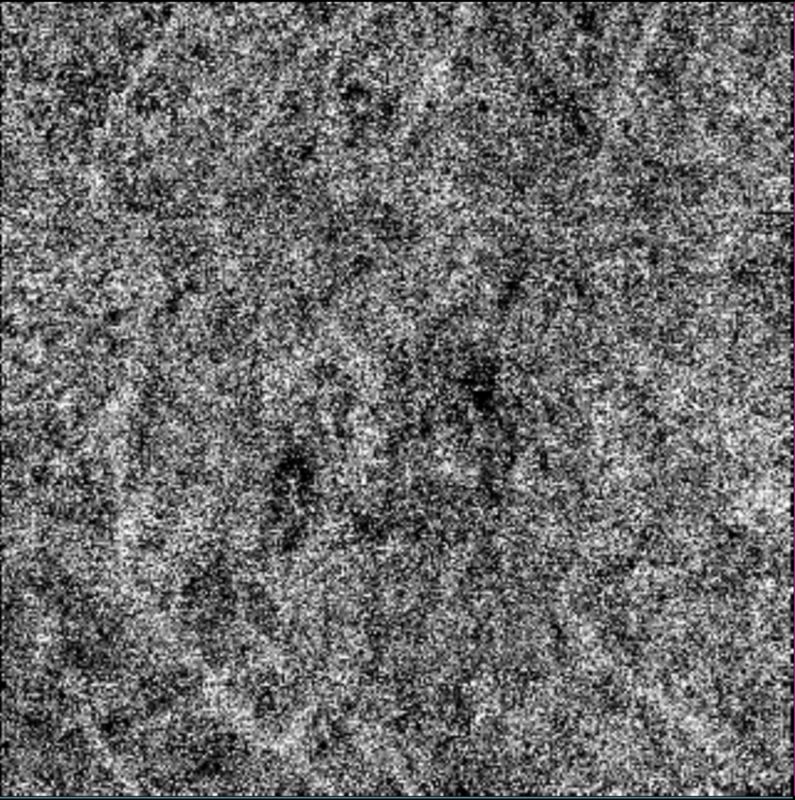

Supplement: S1 File — (ZIP) [file pone.0306735.s002.zip › 17_OD.jpg]

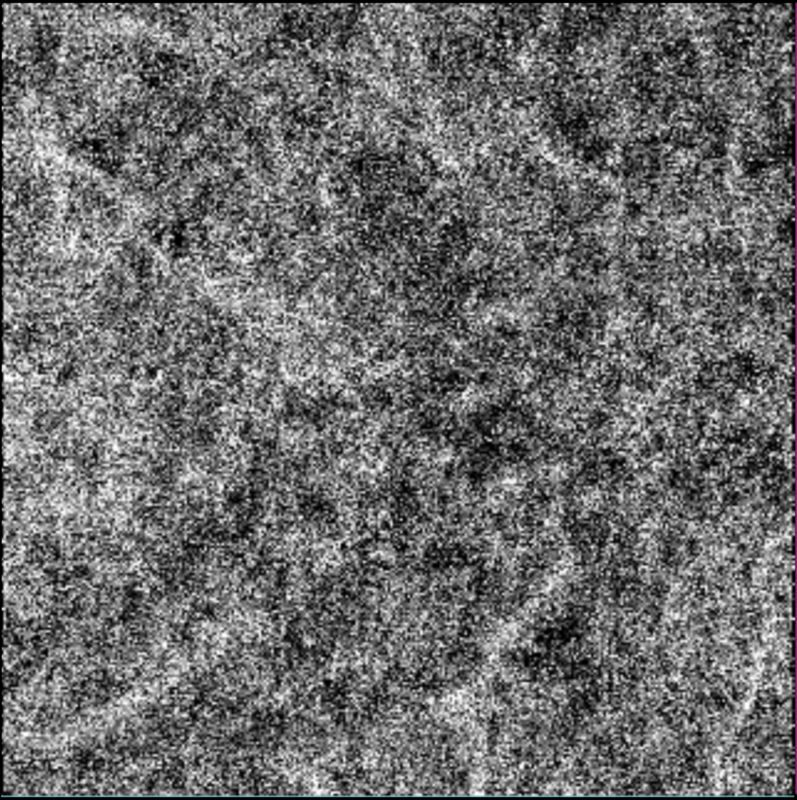

Supplement: S1 File — (ZIP) [file pone.0306735.s002.zip › 17_OS.jpg]

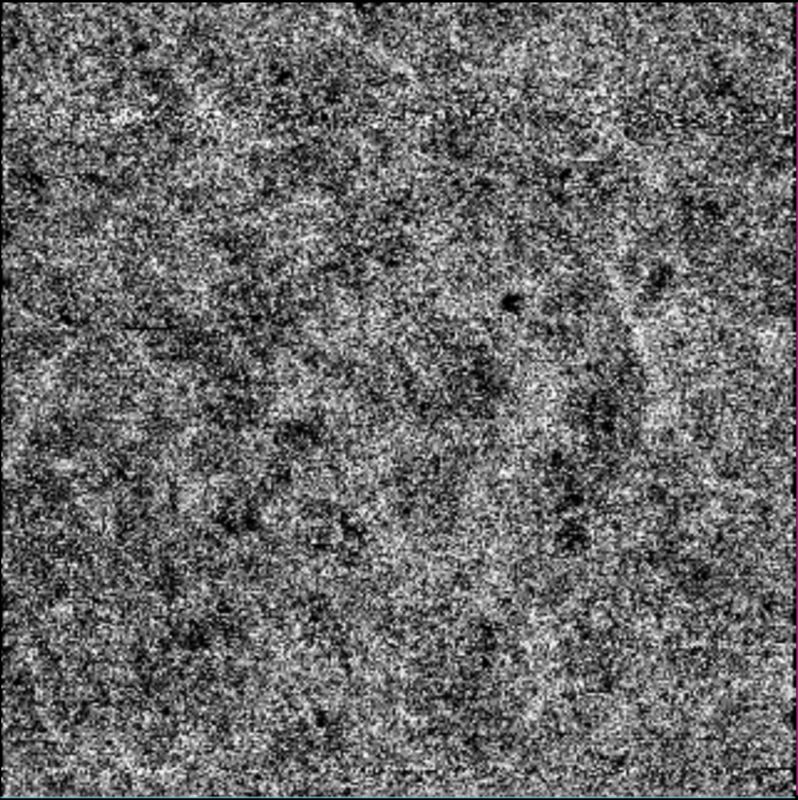

Supplement: S1 File — (ZIP) [file pone.0306735.s002.zip › 18_OD.jpg]

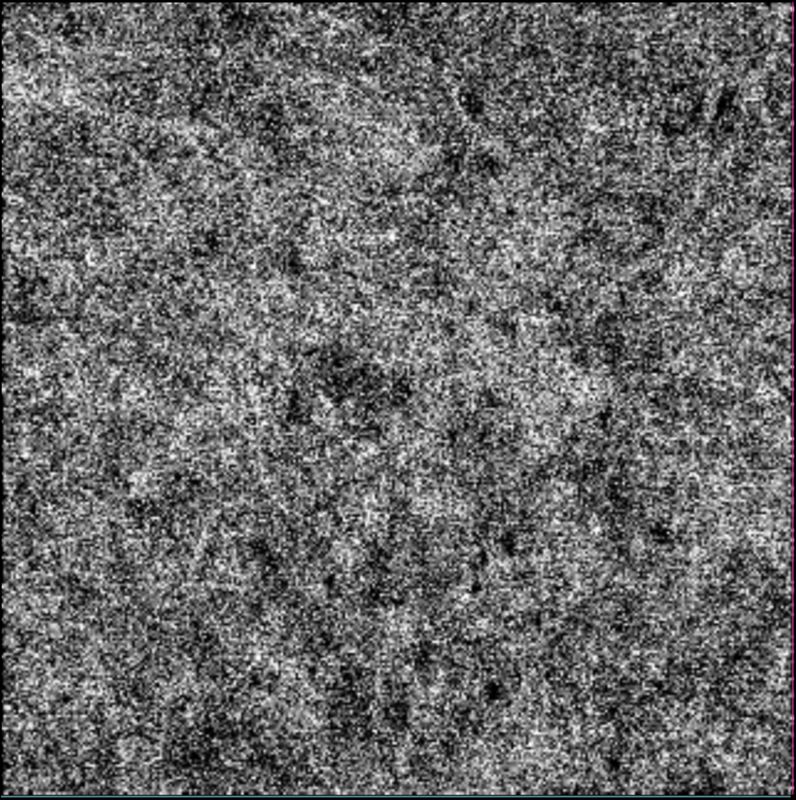

Supplement: S1 File — (ZIP) [file pone.0306735.s002.zip › 18_OS.jpg]

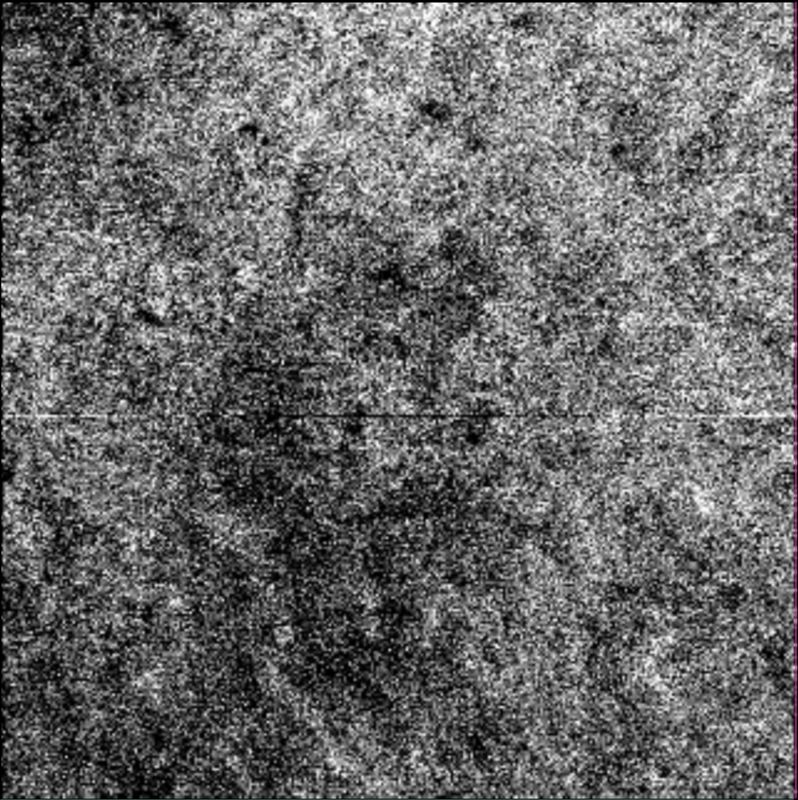

Supplement: S1 File — (ZIP) [file pone.0306735.s002.zip › 19_OD.jpg]

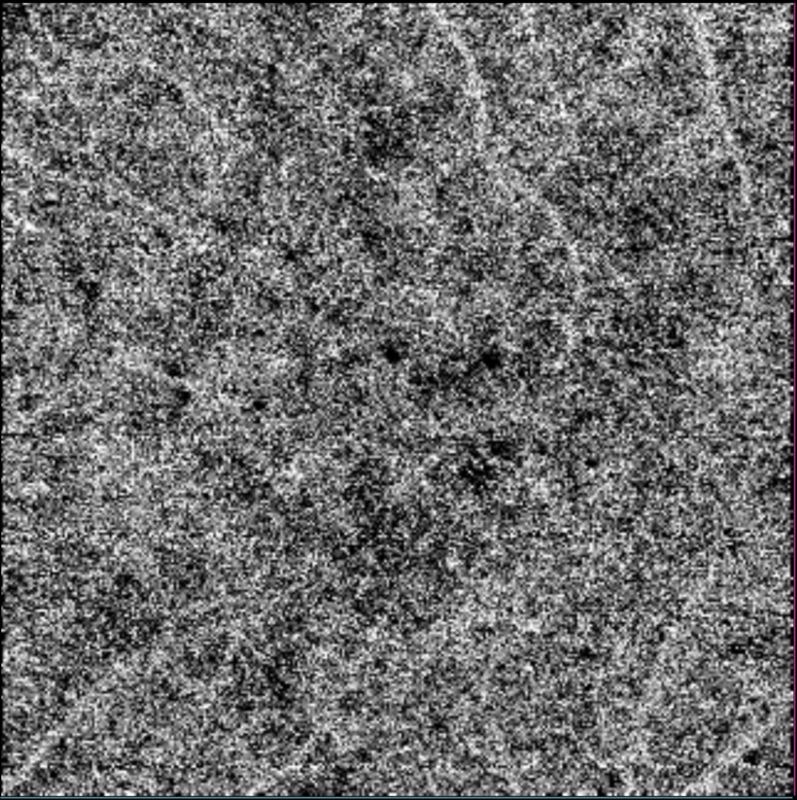

Supplement: S1 File — (ZIP) [file pone.0306735.s002.zip › 19_OS.jpg]

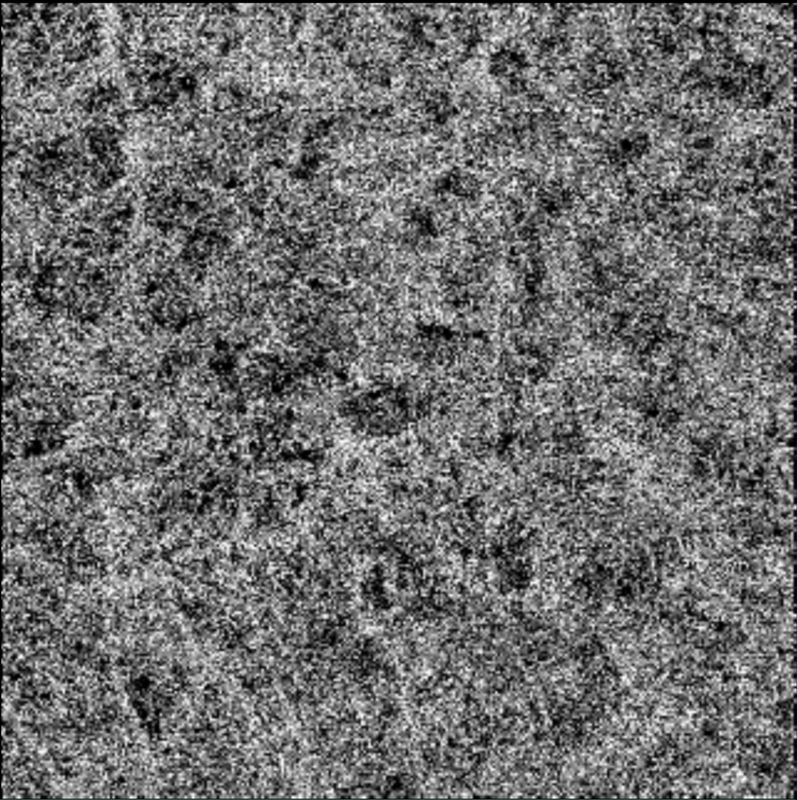

Supplement: S1 File — (ZIP) [file pone.0306735.s002.zip › 1_OD.jpg]

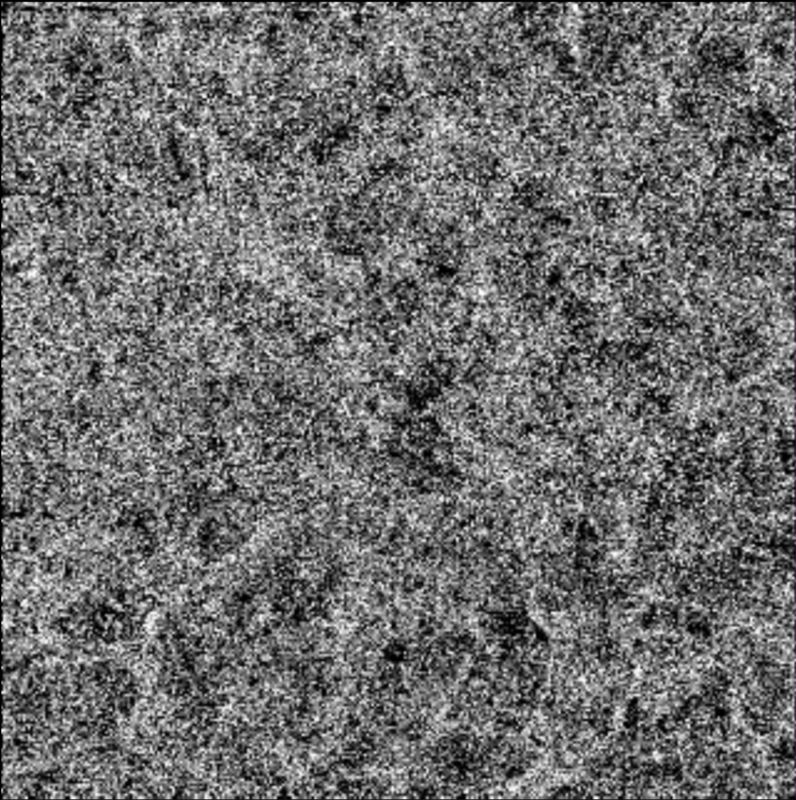

Supplement: S1 File — (ZIP) [file pone.0306735.s002.zip › 1_OS.jpg]

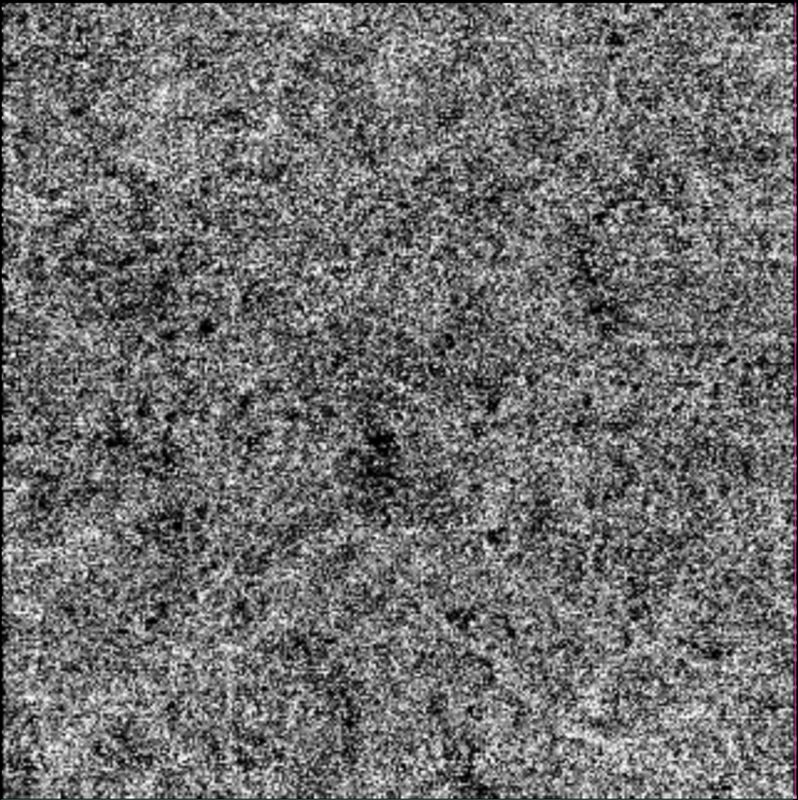

Supplement: S1 File — (ZIP) [file pone.0306735.s002.zip › 20_OD.jpg]

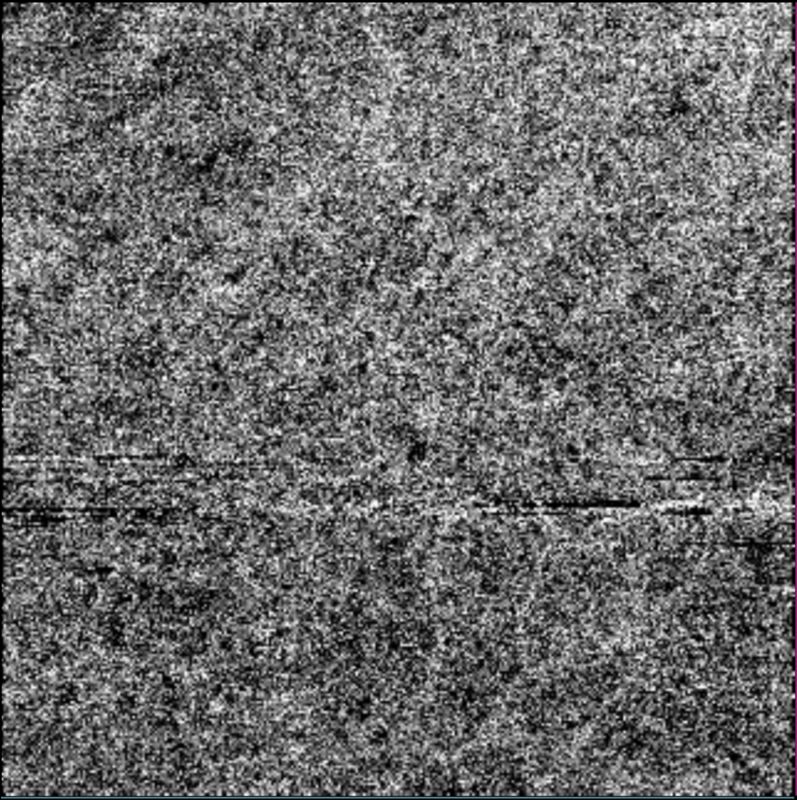

Supplement: S1 File — (ZIP) [file pone.0306735.s002.zip › 20_OS.jpg]

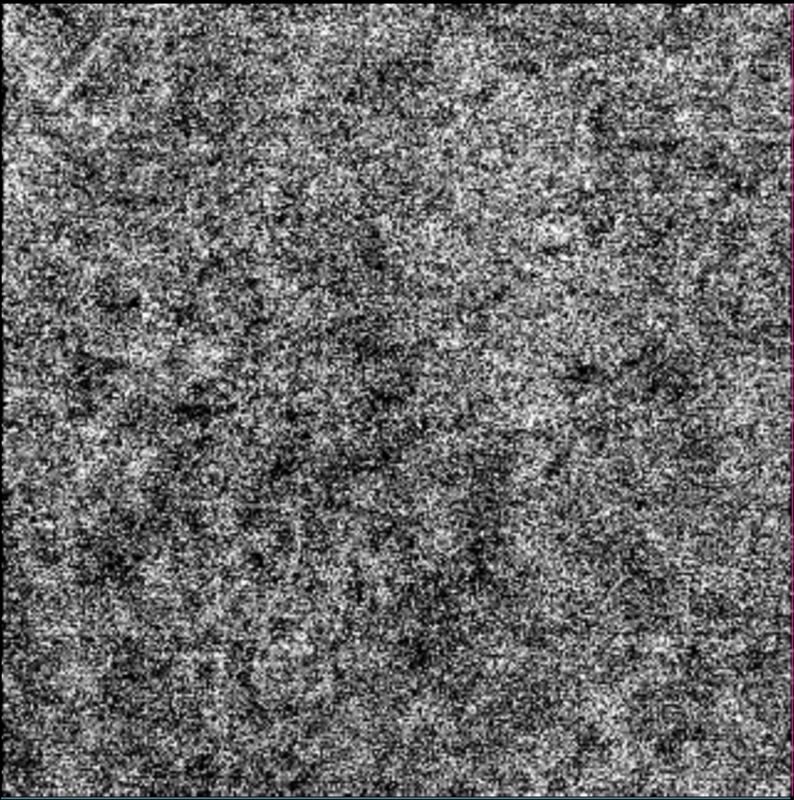

Supplement: S1 File — (ZIP) [file pone.0306735.s002.zip › 21_OD.jpg]

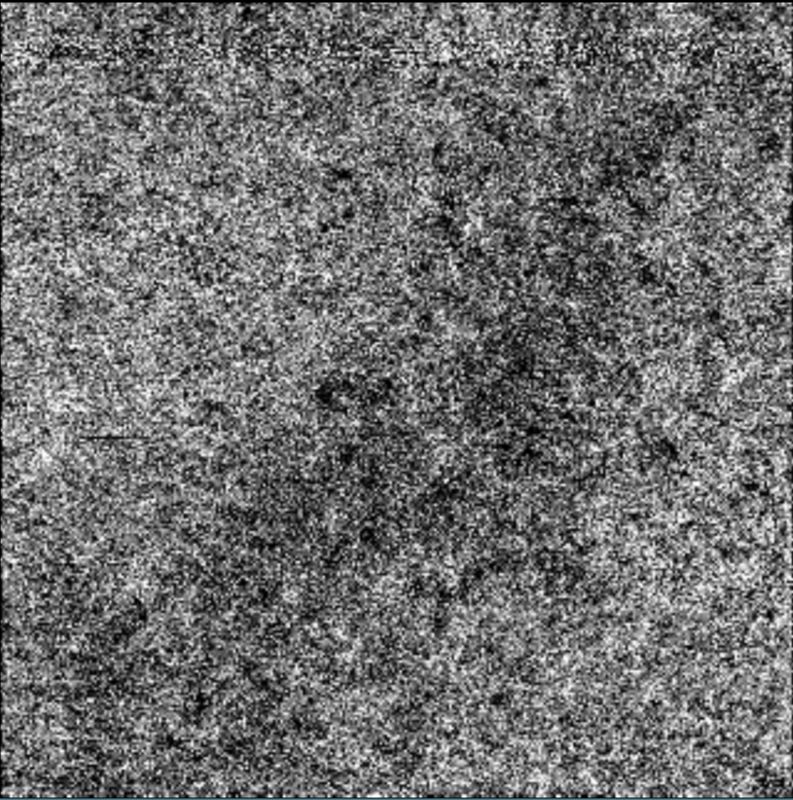

Supplement: S1 File — (ZIP) [file pone.0306735.s002.zip › 21_OS.jpg]

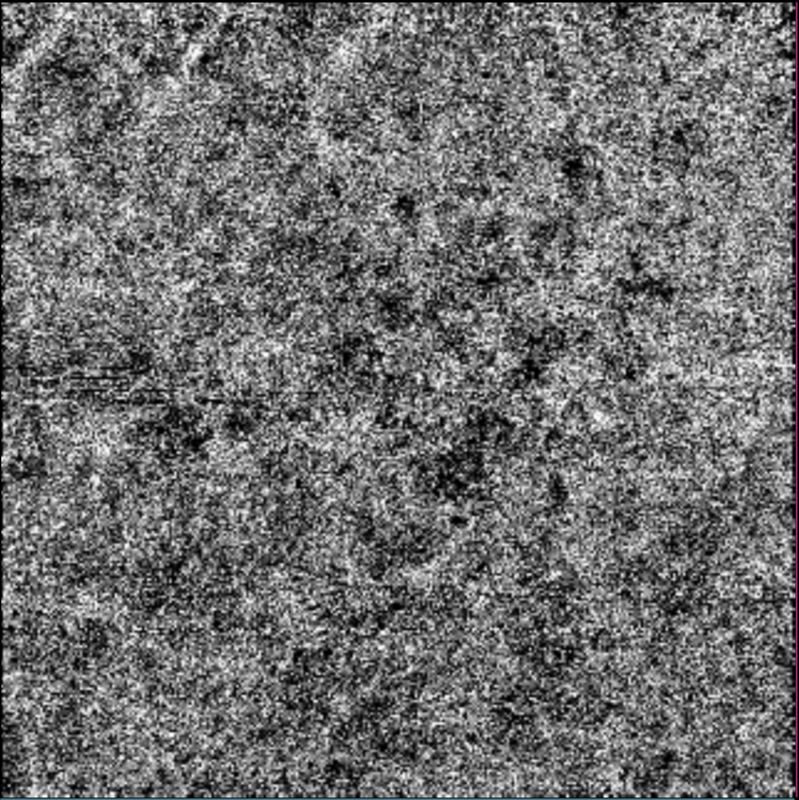

Supplement: S1 File — (ZIP) [file pone.0306735.s002.zip › 22_OD.jpg]

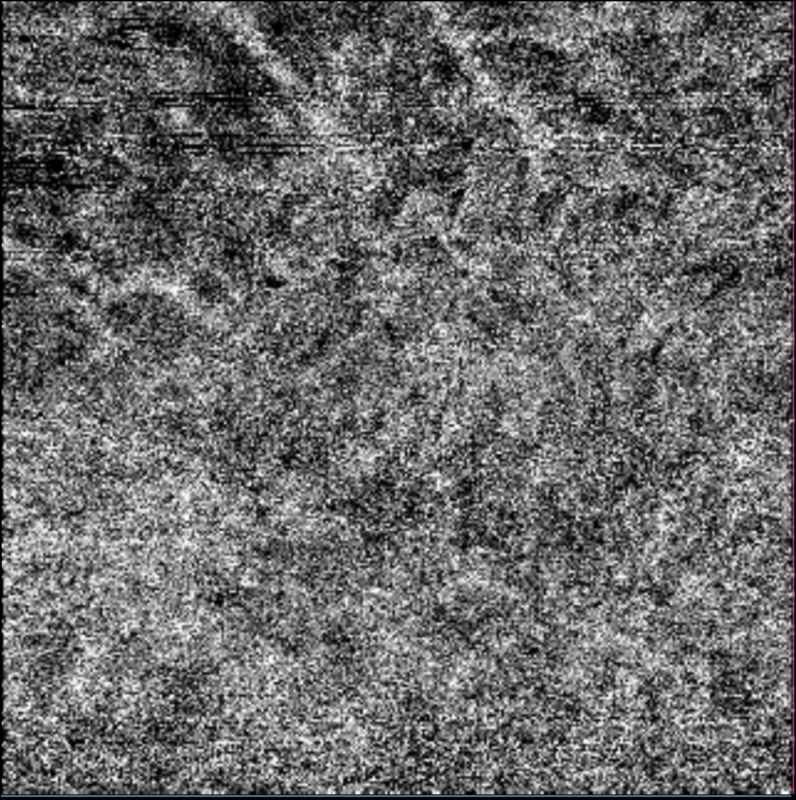

Supplement: S1 File — (ZIP) [file pone.0306735.s002.zip › 22_OS.jpg]

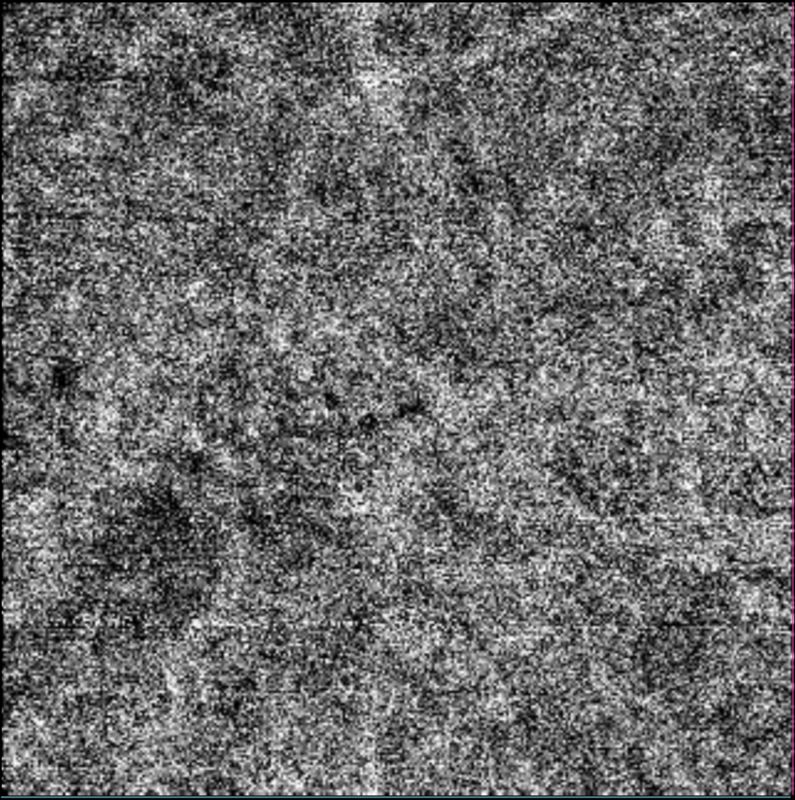

Supplement: S1 File — (ZIP) [file pone.0306735.s002.zip › 23_OD.jpg]

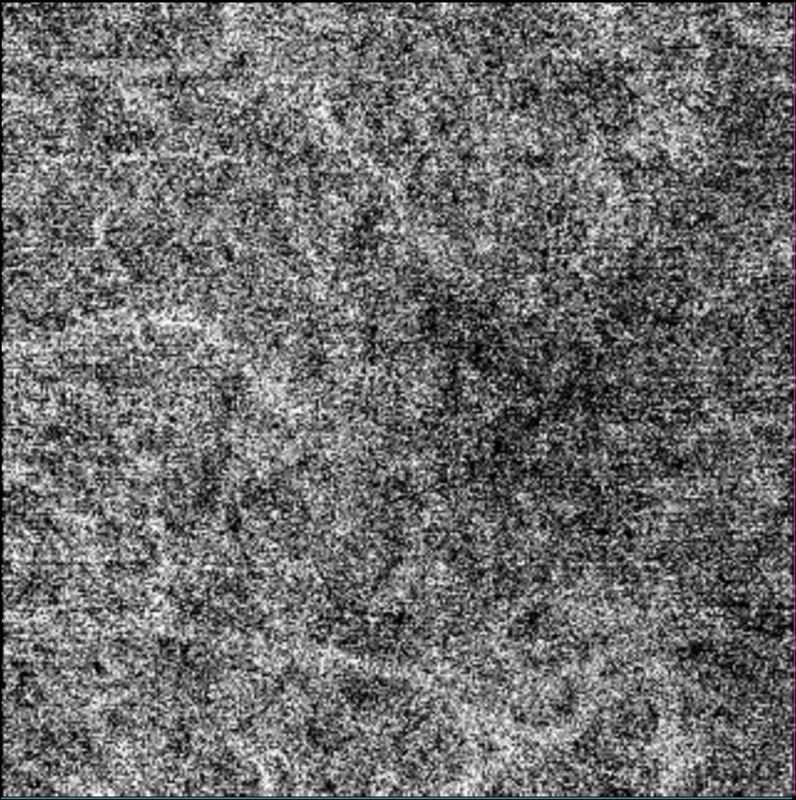

Supplement: S1 File — (ZIP) [file pone.0306735.s002.zip › 23_OS.jpg]

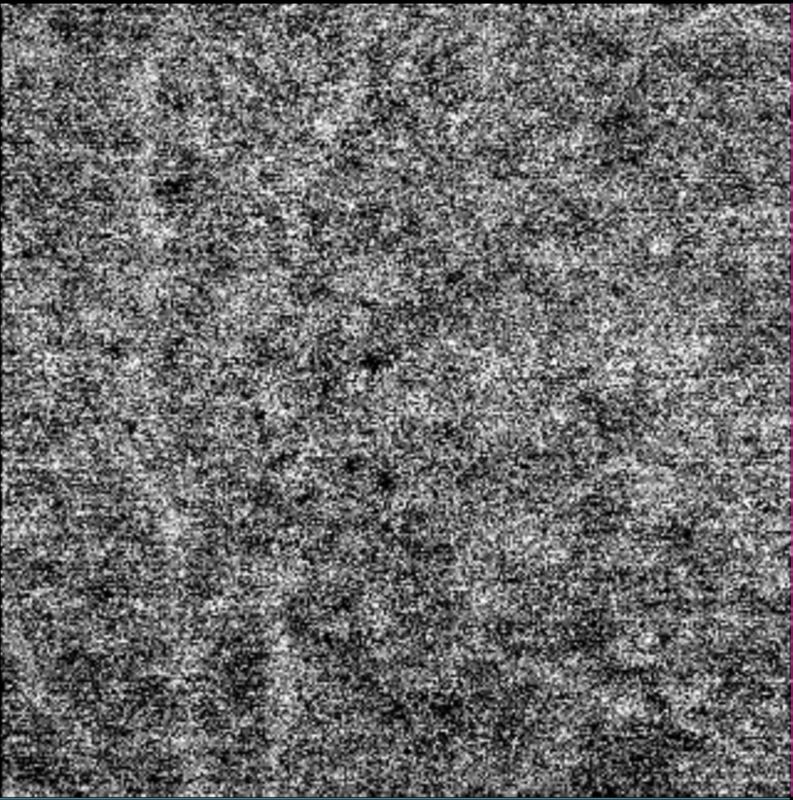

Supplement: S1 File — (ZIP) [file pone.0306735.s002.zip › 24_OD.jpg]

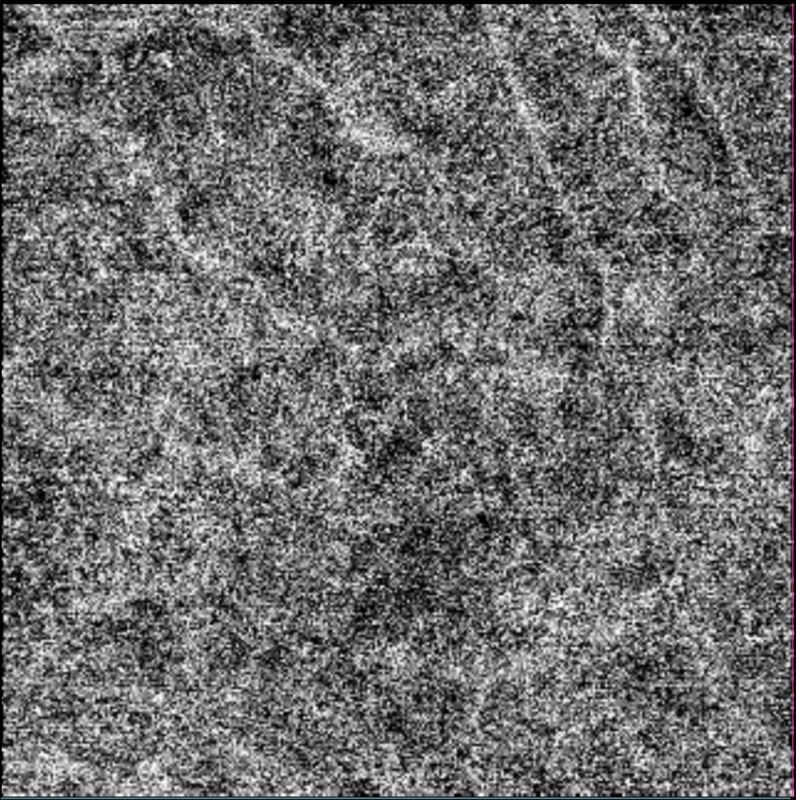

Supplement: S1 File — (ZIP) [file pone.0306735.s002.zip › 24_OS.jpg]

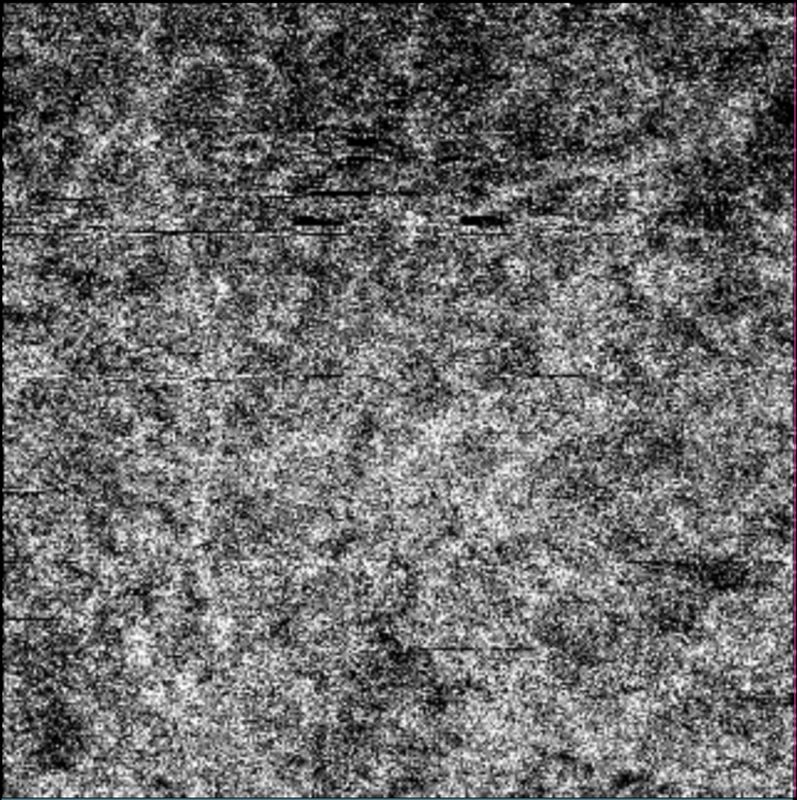

Supplement: S1 File — (ZIP) [file pone.0306735.s002.zip › 25_OD.jpg]

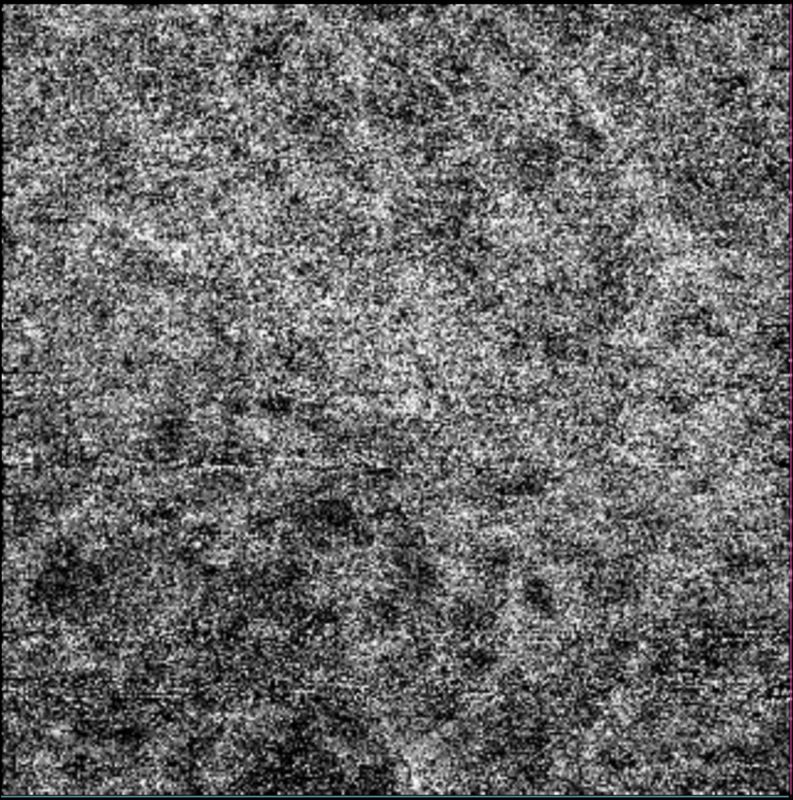

Supplement: S1 File — (ZIP) [file pone.0306735.s002.zip › 25_OS.jpg]

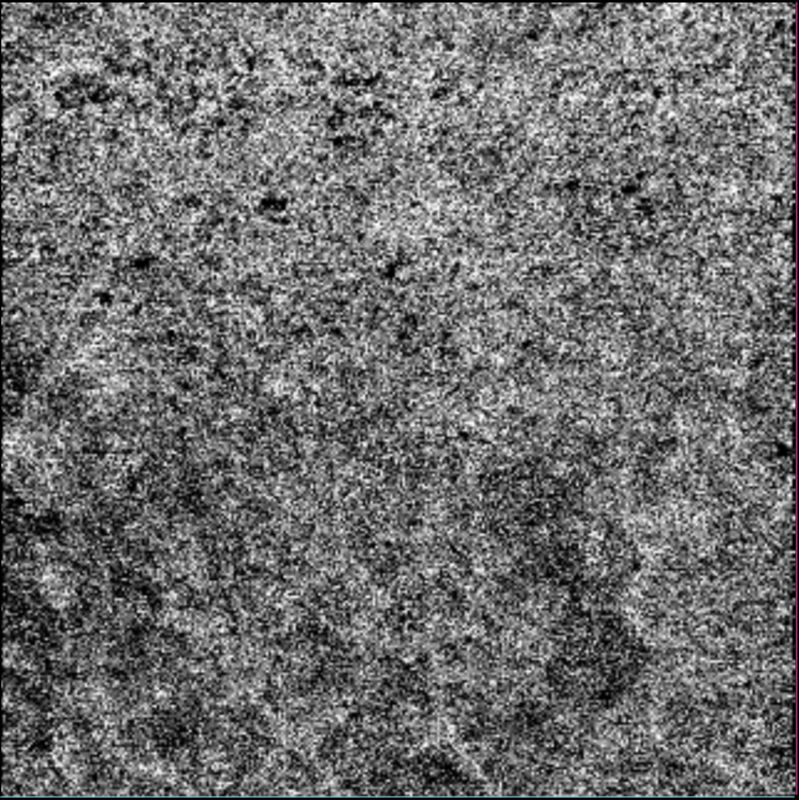

Supplement: S1 File — (ZIP) [file pone.0306735.s002.zip › 26_OD.jpg]

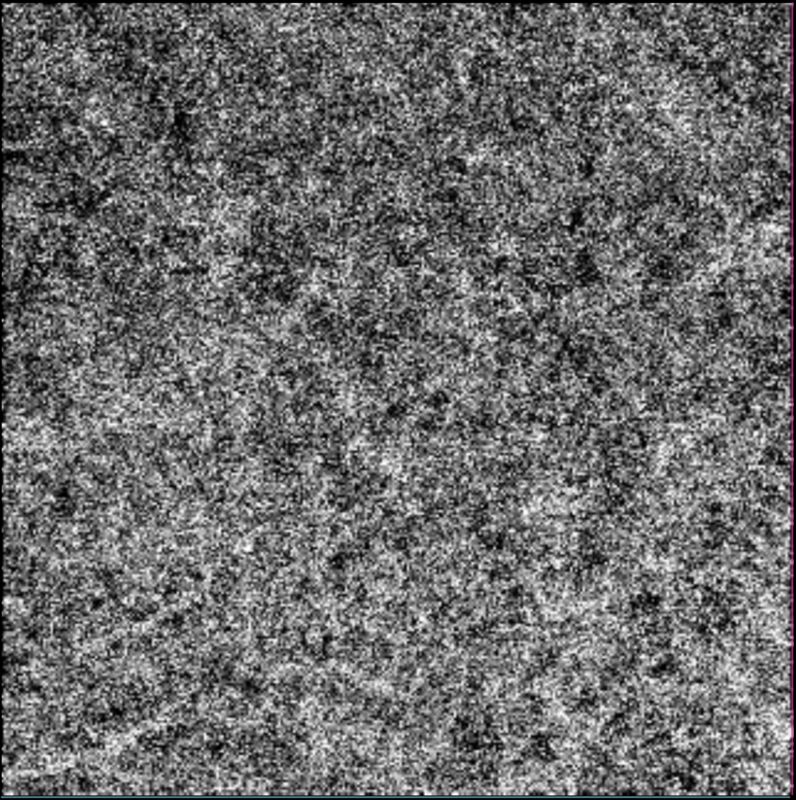

Supplement: S1 File — (ZIP) [file pone.0306735.s002.zip › 26_OS.jpg]

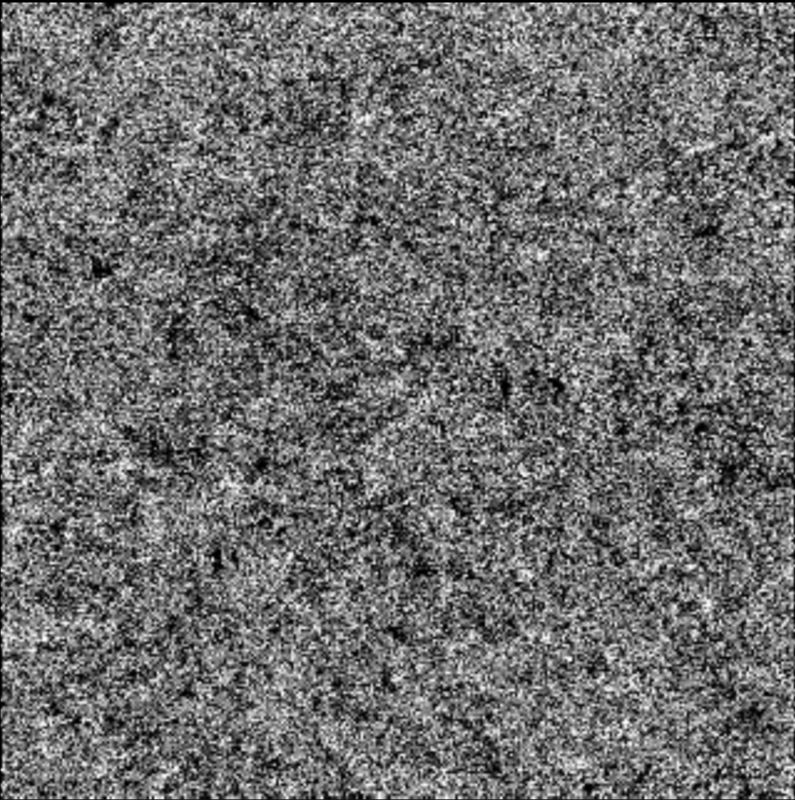

Supplement: S1 File — (ZIP) [file pone.0306735.s002.zip › 27_OD.jpg]

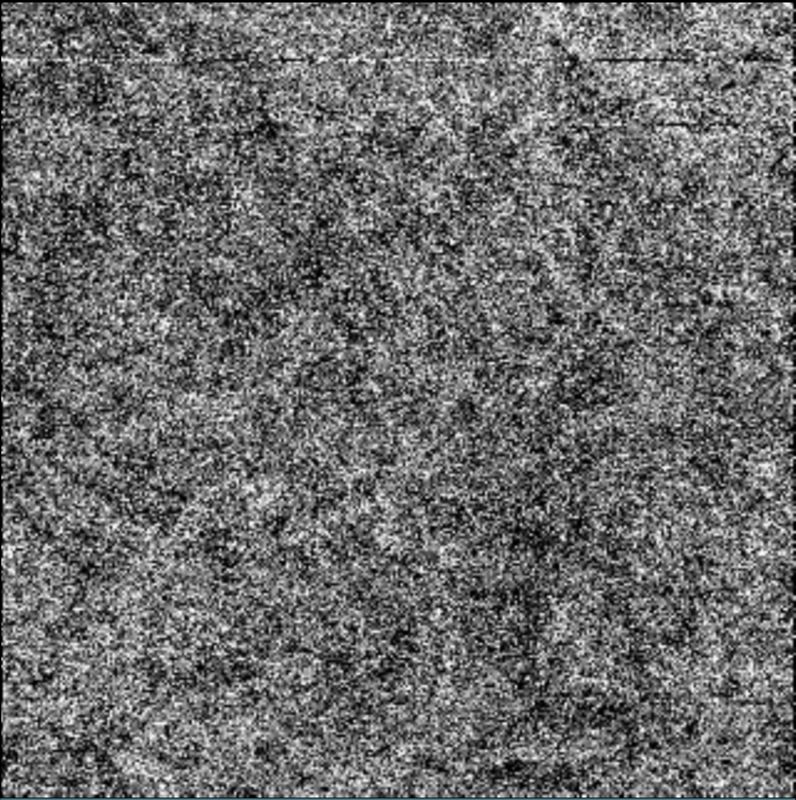

Supplement: S1 File — (ZIP) [file pone.0306735.s002.zip › 27_OS.jpg]

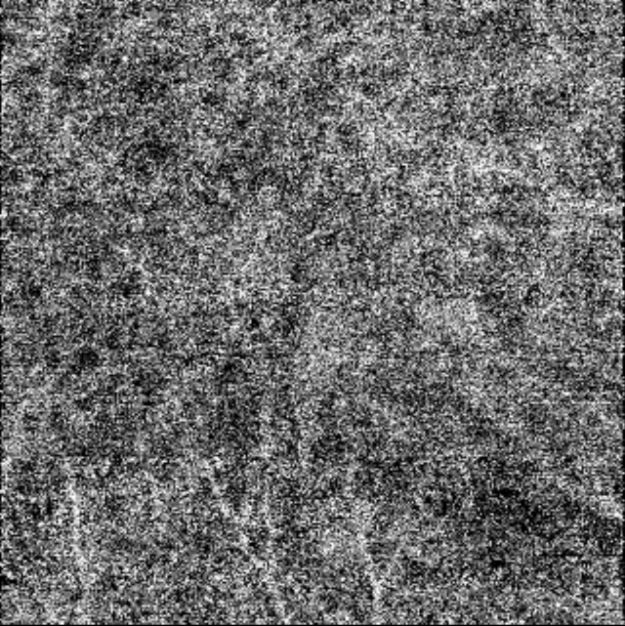

Supplement: S1 File — (ZIP) [file pone.0306735.s002.zip › 28_OD.jpg]

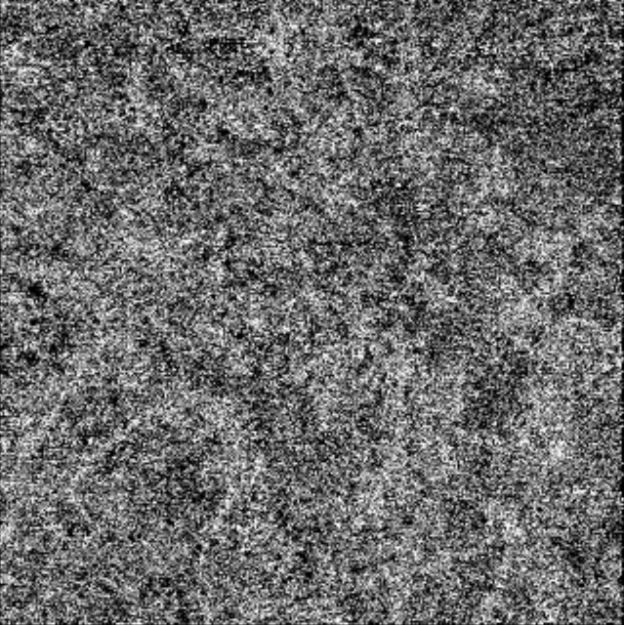

Supplement: S1 File — (ZIP) [file pone.0306735.s002.zip › 28_OS.jpg]

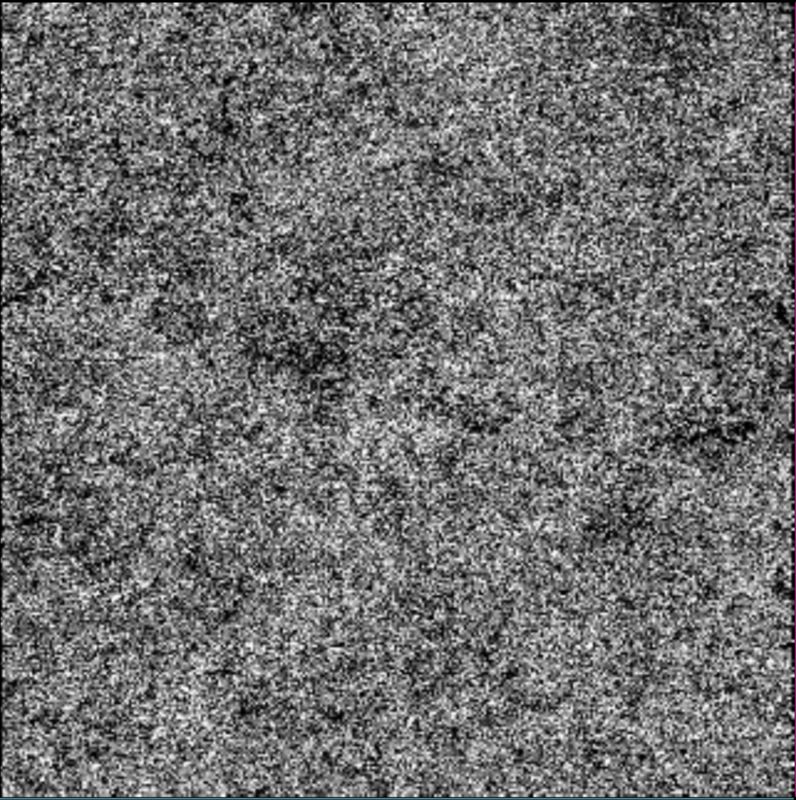

Supplement: S1 File — (ZIP) [file pone.0306735.s002.zip › 2_OD.jpg]

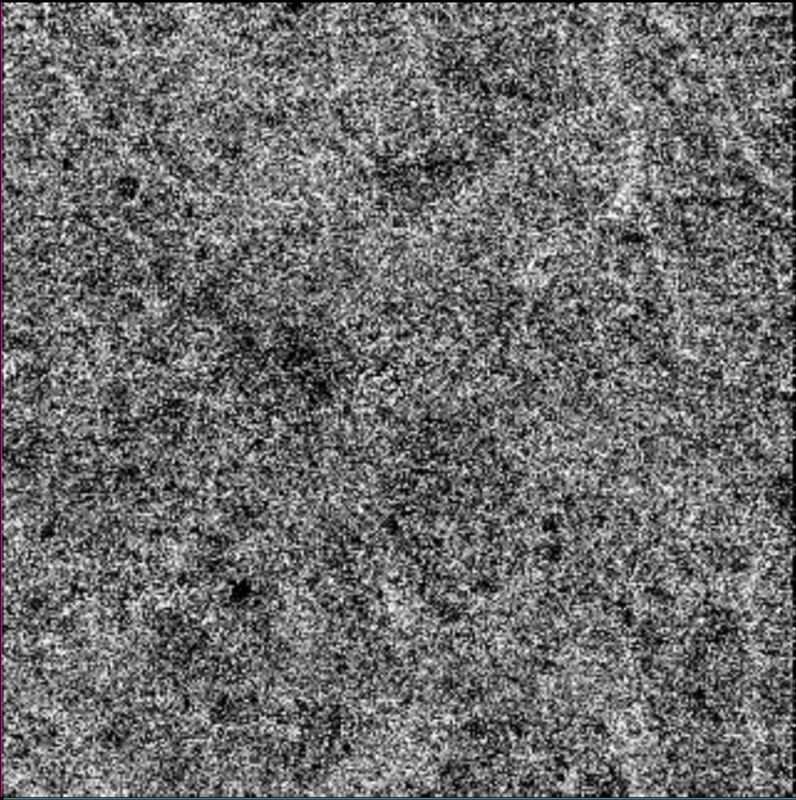

Supplement: S1 File — (ZIP) [file pone.0306735.s002.zip › 2_OS.jpg]

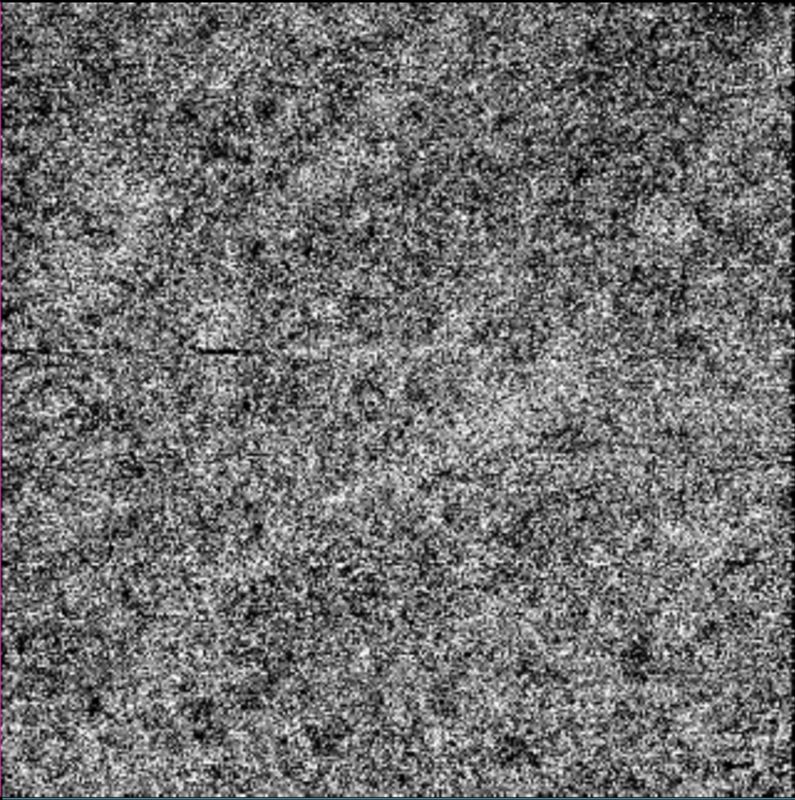

Supplement: S1 File — (ZIP) [file pone.0306735.s002.zip › 3_OD.jpg]

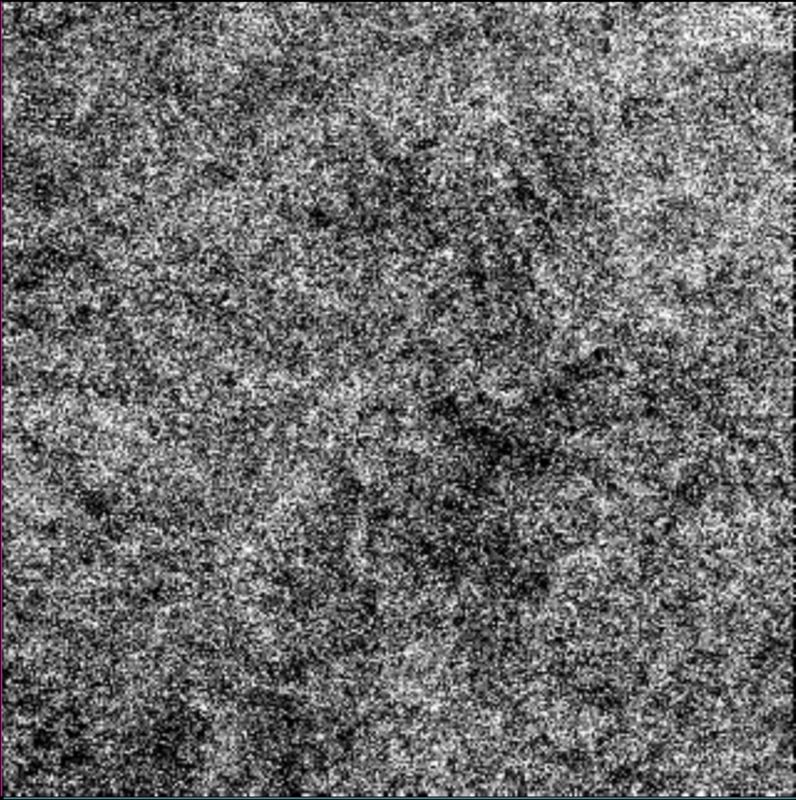

Supplement: S1 File — (ZIP) [file pone.0306735.s002.zip › 3_OS.jpg]

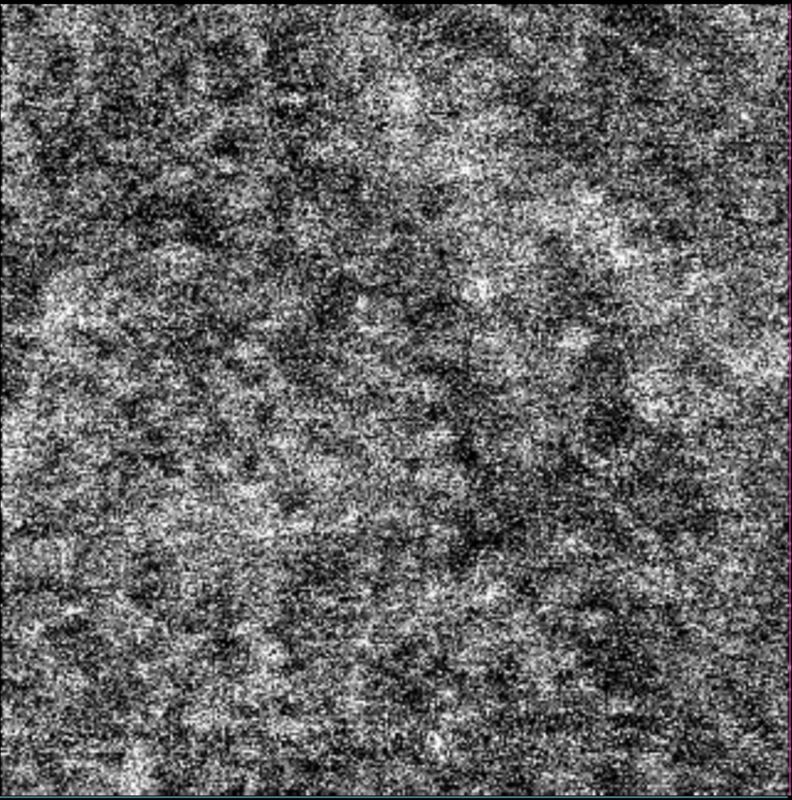

Supplement: S1 File — (ZIP) [file pone.0306735.s002.zip › 4_OD.jpg]

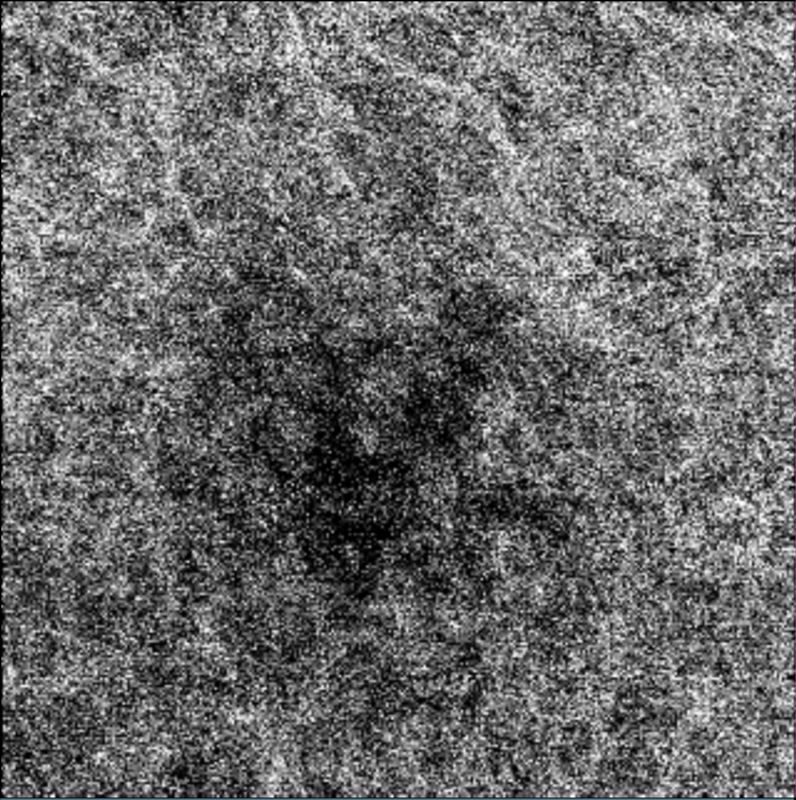

Supplement: S1 File — (ZIP) [file pone.0306735.s002.zip › 4_OS.jpg]

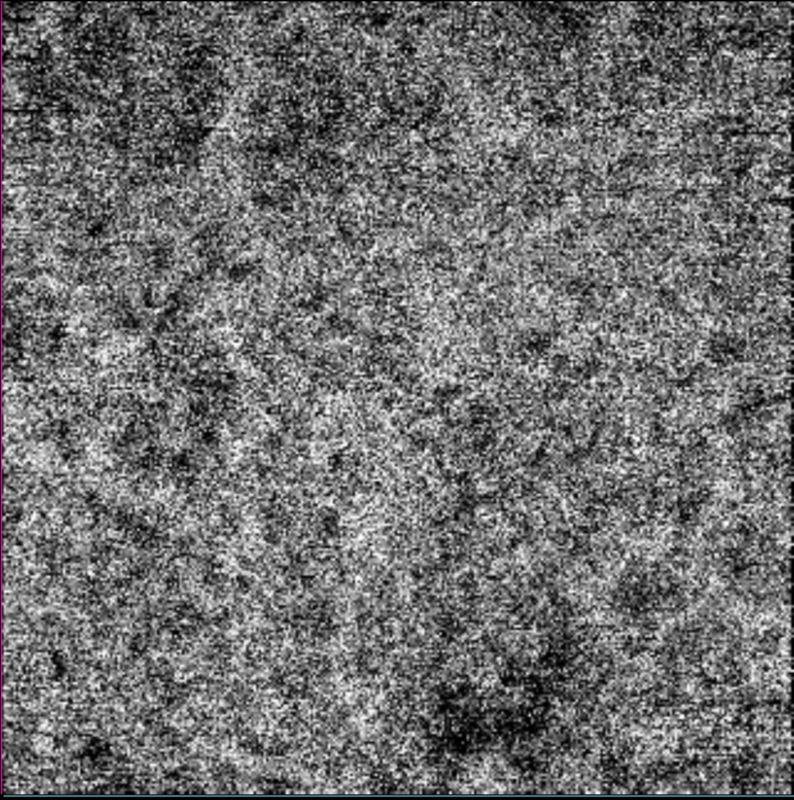

Supplement: S1 File — (ZIP) [file pone.0306735.s002.zip › 5_OD.jpg]

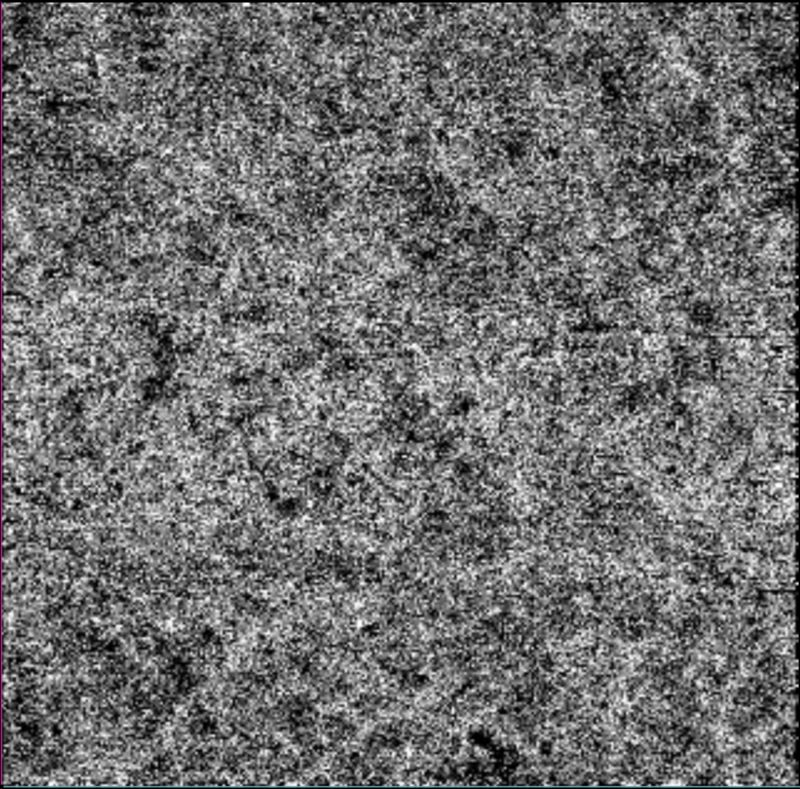

Supplement: S1 File — (ZIP) [file pone.0306735.s002.zip › 5_OS.jpg]

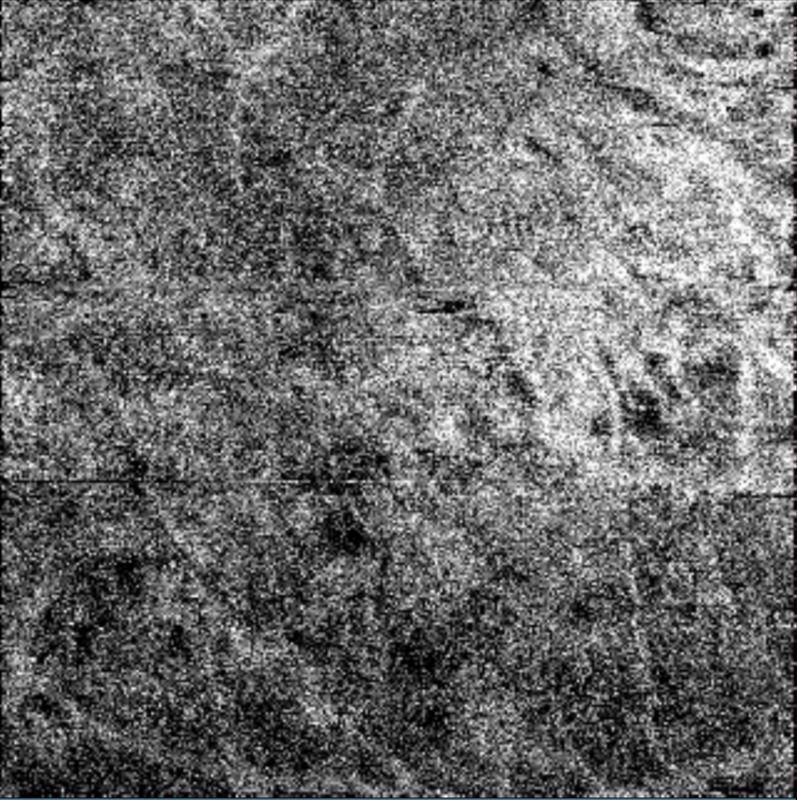

Supplement: S1 File — (ZIP) [file pone.0306735.s002.zip › 6_OD.jpg]

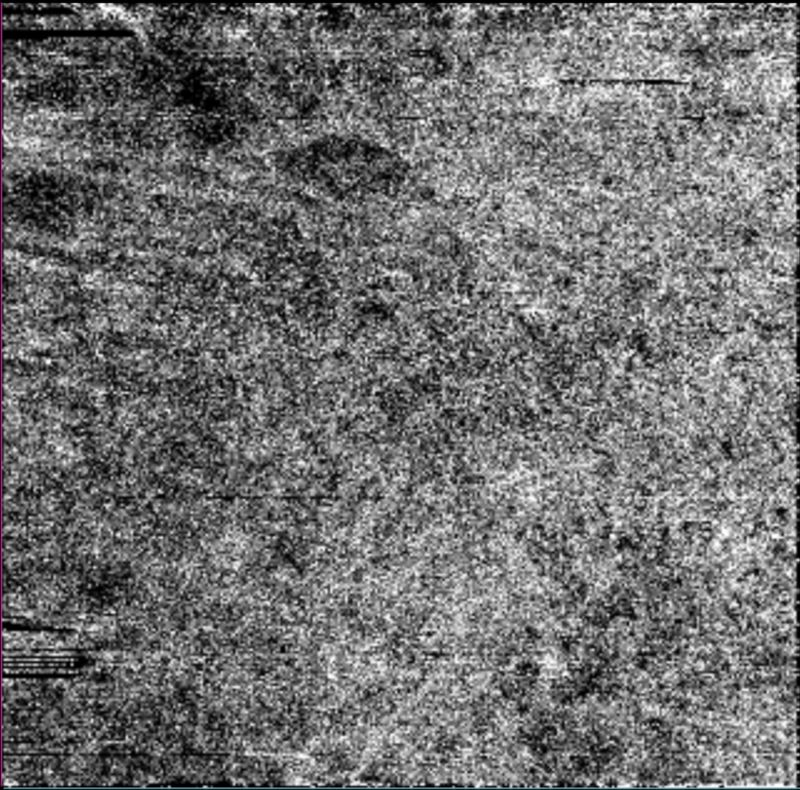

Supplement: S1 File — (ZIP) [file pone.0306735.s002.zip › 6_OS.jpg]

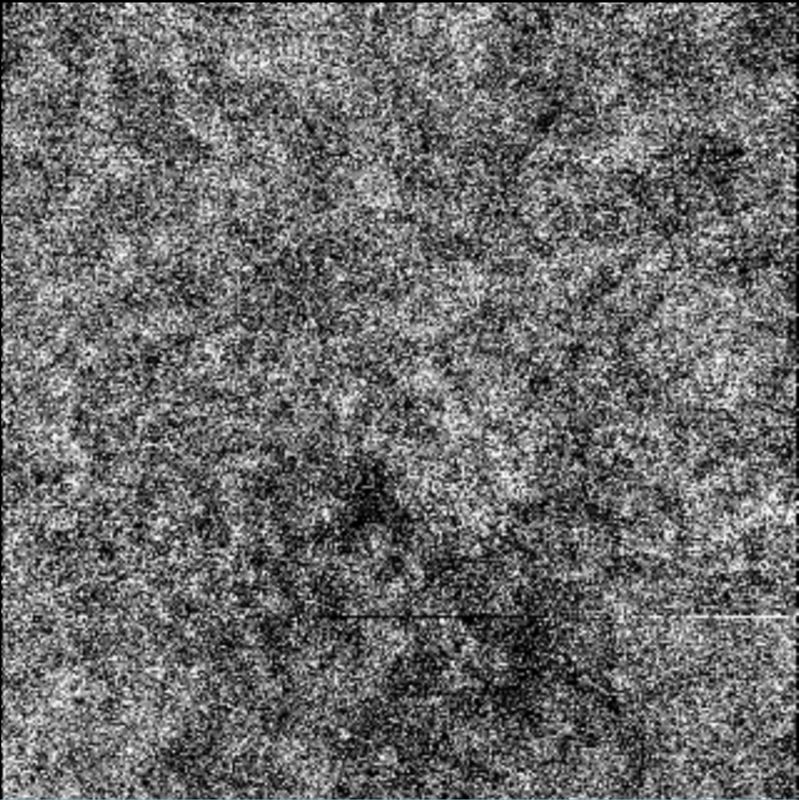

Supplement: S1 File — (ZIP) [file pone.0306735.s002.zip › 7_OD.jpg]

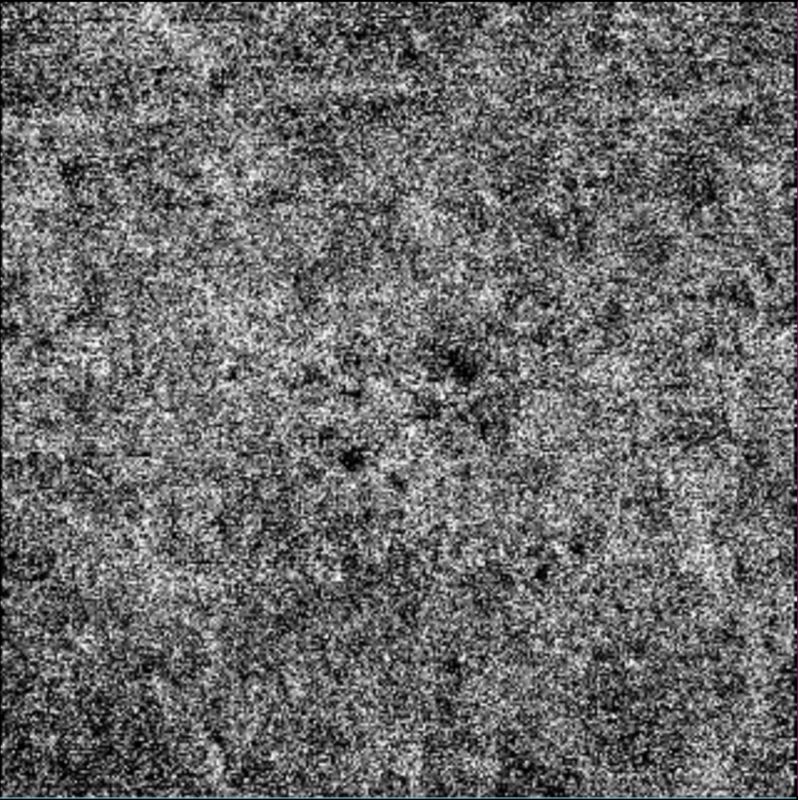

Supplement: S1 File — (ZIP) [file pone.0306735.s002.zip › 7_OS.jpg]

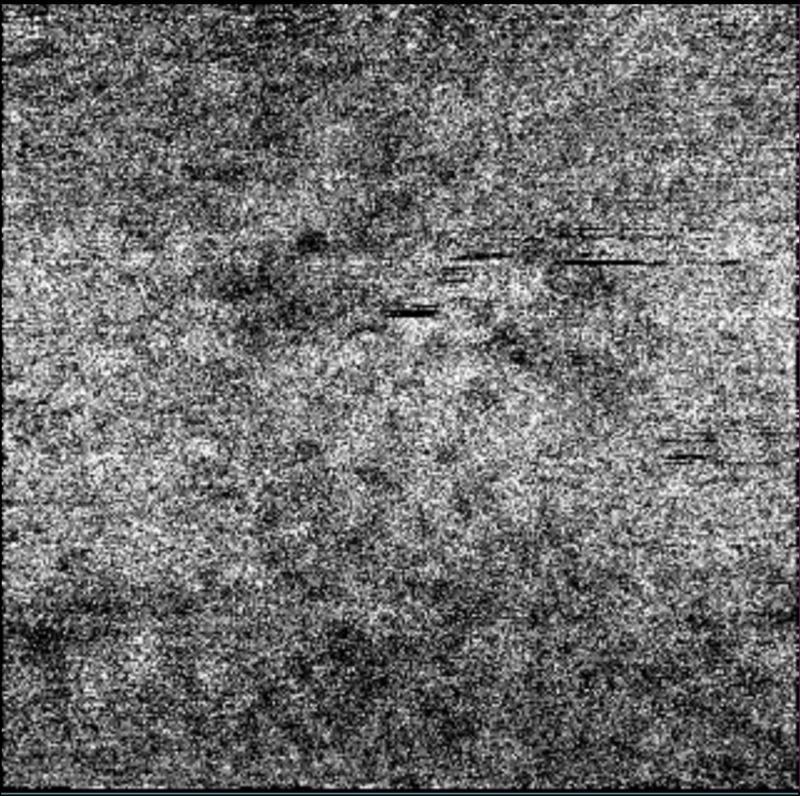

Supplement: S1 File — (ZIP) [file pone.0306735.s002.zip › 8_OD.jpg]

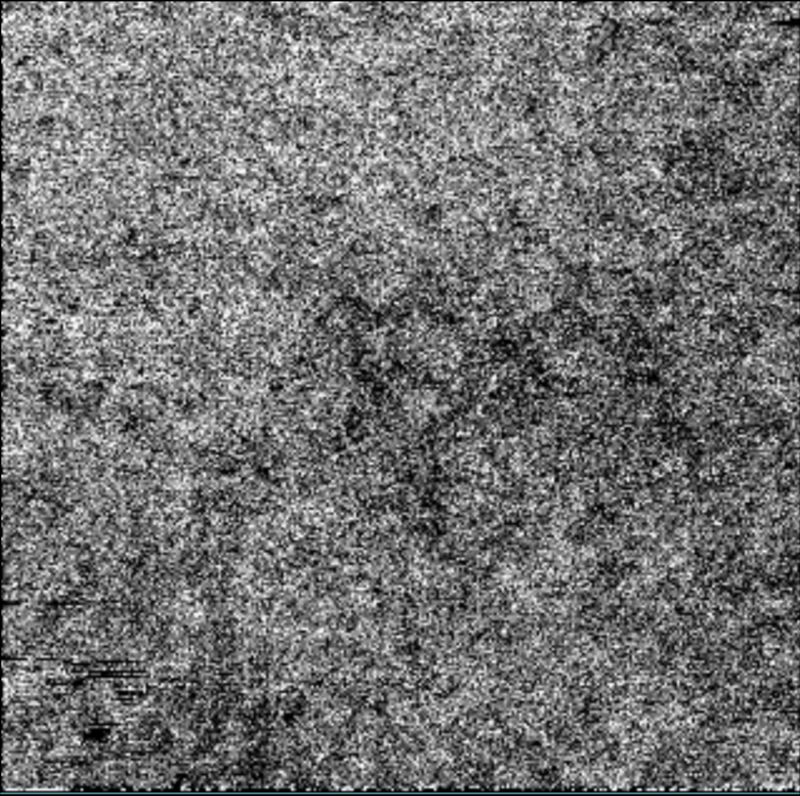

Supplement: S1 File — (ZIP) [file pone.0306735.s002.zip › 8_OS.jpg]

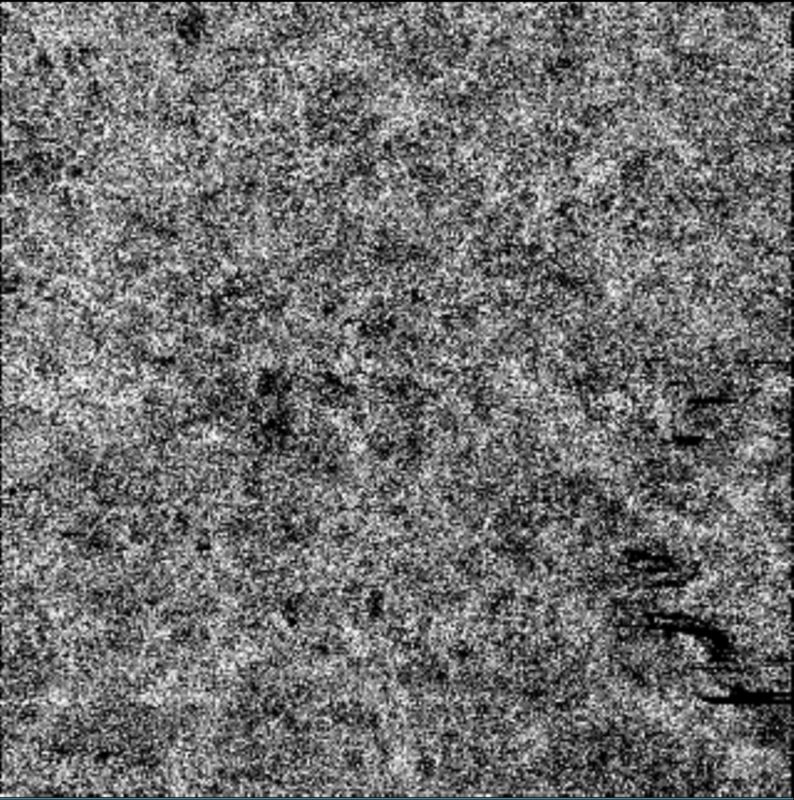

Supplement: S1 File — (ZIP) [file pone.0306735.s002.zip › 9_OD.jpg]

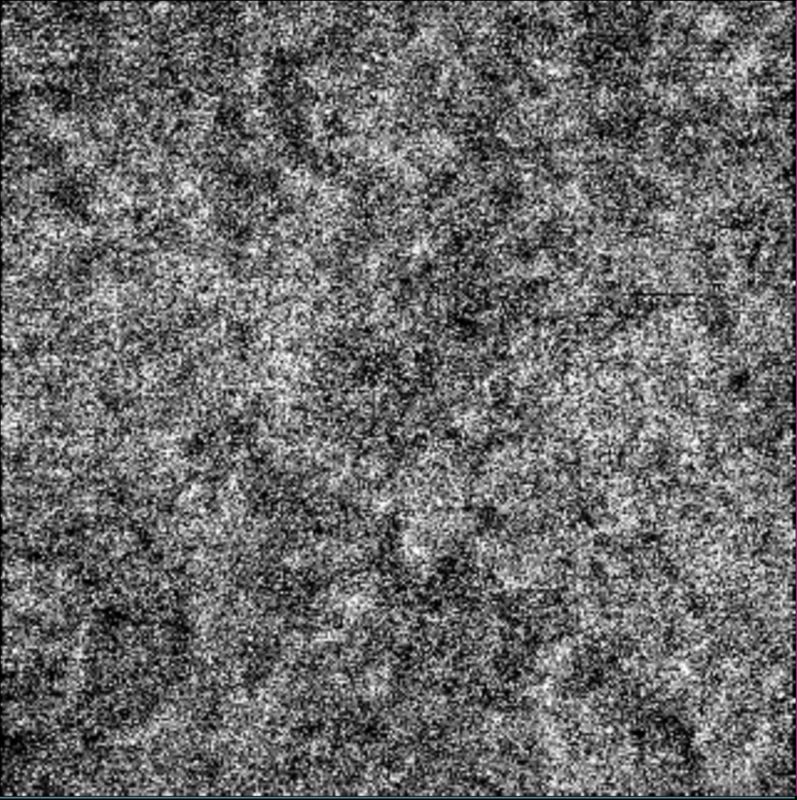

Supplement: S1 File — (ZIP) [file pone.0306735.s002.zip › 9_OS.jpg]
